# Current smoking and COPD are associated with differentiation-dependent secretory and inflammatory programs in airway basal cells

**DOI:** 10.1186/s12931-026-03847-4

**Published:** 2026-08-29

**Authors:** Felix Ritzmann, Michelle Brand, Gilles Gasparoni, Xuan Zhang, Frank Langer, Christian Herr, Yiwen Yao, Migdat Mustafi, Daniela Yildiz, Jörn Walter, Robert Bals, Christoph Beisswenger

**Affiliations:** 1 Department of Internal Medicine V—Pulmonology, Allergology and Respiratory Critical Care Medicine, Saarland University, Homburg, 66421 Germany; 2 Microbial Natural Products (MINS), Helmholtz-Institute for Pharmaceutical Research Saarland (HIPS), Helmholtz Centre for Infection Research (HZI), Saarland University Campus, Saarbrücken, 66123 Germany; 3 Department of Genetics, Saarland University, Saarbrücken, Germany; 4 Department of Thoracic Surgery, Saarland University Hospital, Homburg/Saar, Germany; 5 Molecular Pharmacology, Center for Molecular Signaling (PZMS), Center for Gender-specific Biology and Medicine (CGBM), Pharma Science Hub (PSH), Saarland University, Homburg, 66421 Germany

**Keywords:** Epigenetics, Lung organoids, COPD, Smoke, Epithelial cells

## Abstract

**Background:**

Persistent airway epithelial abnormalities contribute to chronic obstructive pulmonary disease (COPD), but it remains unclear whether smoking- and COPD-associated epithelial remodeling is retained in airway basal cells and transmitted during differentiation. We determined whether current smoking and COPD are associated with methylation-linked regulatory programs in airway basal cells that shape epithelial differentiation in patient-derived bronchial organoids.

**Methods:**

We integrated DNA methylation profiling and bulk transcriptomics in patient-derived airway basal cells and matched three-dimensional bronchial organoids. Methylation-defined gene sets were mapped to organoid epithelial cell states using single-cell RNA-seq and contextualized with publicly available airway epithelial datasets.

**Results:**

In this exploratory cohort, current smoking was associated with a predominant shift toward promoter hypomethylation in airway basal cells and matched organoids. Hypomethylated promoters were enriched for genes preferentially expressed in secretory epithelial cells, including *BPIFB1*, *BPIFA2*, *MSMB* and *GALNT6*. These genes showed little smoking-associated expression difference in basal-cell culture but were upregulated after organoid differentiation, indicating a differentiation-dependent epithelial memory of smoking. In COPD-derived basal cells, promoter methylation changes involved reduced xenobiotic metabolism and enhanced immune- and infection-related programs. Consistently, COPD-derived organoids showed reduced expression of detoxification-associated pathways and increased lysosomal, endocytic and host-defense programs.

**Conclusions:**

Current smoking and COPD are associated with persistent methylation-linked regulatory alterations in airway basal cells that become functionally apparent during epithelial differentiation. These findings support a model in which airway basal-cell memory contributes to secretory, inflammatory and host-defense remodeling in chronic airway disease.

**Supplementary Information:**

The online version contains supplementary material available at https://doi.org/10.1186/s12931-026-03847-4.

## Introduction

Chronic obstructive pulmonary disease (COPD) is a progressive lung disorder characterized by chronic inflammation, pathological airway remodeling and parenchymal destruction leading to emphysema. Cigarette smoking is the major risk factor for COPD and induces persistent airway inflammation as well as structural changes in the airway epithelium. These include goblet cell metaplasia, mucus hypersecretion and ciliary dysfunction, resulting in impaired mucociliary clearance. Airway remodeling in COPD further involves small-airway wall thickening, fibrosis and alterations of the extracellular matrix [1–3]. Together, these changes contribute to airflow limitation and loss of gas exchange surface [4]. However, it remains unclear whether epithelial abnormalities in smokers and patients with COPD primarily reflect ongoing exposure and inflammation, or whether they are retained within the airway basal-cell compartment and re-emerge during epithelial differentiation.

The epigenome plays a central role in development and in mature organisms, regulating processes such as the maintenance of stem cells and lineage-specific differentiation [5, 6]. Environmental factors can influence the genome through epigenetic modifications, with cigarette smoking representing a major factor that likely alters the epigenome in multiple compartments [6]. There is evidence that smoking is associated with DNA hypomethylation in blood cells, although many of these epigenetic changes can revert following smoking cessation [7–9]. For example, differential methylation of the aryl-hydrocarbon receptor repressor (*AHRR*) has been consistently linked to smoking, and longitudinal studies have shown that methylation at the cg05575921 site in the *AHRR* gene can recover after cessation, illustrating the dynamic nature of smoking-induced epigenetic modifications [10]. A systematic review of genome-wide DNA methylation studies highlighted several genes associated with COPD and lung function. *AHRR* methylation was linked, for instance, to both spirometric parameters and COPD, while *RUNX3* methylation associated with longitudinal changes in FEV1 and FVC [9]. Other findings involved genes related to immune response, erythrocyte differentiation, and cytokine production, suggesting that epigenetic alterations influence key pathways relevant to COPD pathogenesis [6, 9, 11].

There is evidence that smoking and COPD are also associated with epigenetic modifications that dysregulate multiple cellular processes and signaling pathways in lung epithelial cells. Analyses of bronchial brushings and lung tissue suggest that smoking and COPD are associated with numerous differentially methylated sites in genes involved in detoxification pathways, xenobiotic metabolism, aryl hydrocarbon receptor signaling, and oxidative stress responses [12–17]. Nevertheless, it remains largely unclear how epigenetic changes in specific compartments, such as the airway epithelium, are linked to smoking and COPD, and how they contribute to the pathogenesis of the disease [6, 11].

Here, we performed integrated DNA methylation and transcriptomic profiling of patient-derived airway basal cells and their differentiated 3D bronchial organoids from individuals with and without COPD to dissect how smoking and COPD reprogram epithelial gene regulation. We mapped differentially methylated genes to specific epithelial cell types using single-cell RNA sequencing of organoids and native airway epithelium. Expression of these genes was further assessed in independent published airway brush-biopsy cohorts and whole-lung homogenates.

## Results

### Current smoking-associated promoter hypomethylation marks secretory epithelial programs in airway basal cells

Airway epithelial basal cells were obtained via brush biopsies during bronchoscopy and cultured in conventional 2D conditions for epigenetic analysis. The study included 5 current non-smokers (2 never smokers, 3 ex-smokers) and 8 current smokers (Table 1, Fig. S1).

**Table 1:** Patient Characteristics

Disease	Smoker status	Pack years	FEV1	FEV1% (predicted)	Gender	Age
COPD	Ex-smoker^1^	(30)	1,61	73,93	Female	61
COPD	Ex-smoker^2^	(23)	1,25	50,65	Male	71
COPD	Smoker	60	1,96	72,17	Male	74
COPD	Smoker	50	1,46	53,67	Female	70
COPD	Smoker	30	0,48	38,95	Female	63
COPD	Smoker	90	0,96	62,33	Male	71
COPD	Ex-smoker^3^	(50)	0,61	23	Female	60
No lung disease	Never	0	1,32	75,63	Female	87
Lung cancer	Never	0	3,07	97,45	Male	70
Lung cancer	Smoker	80	2,56	81,79	Male	76
Lung cancer, CPFE	Smoker	50	3,2	94,2	Male	75
Lung cancer	Smoker	25	ND	ND	Male	73
Lung cancer	Smoker	30	2,3	73,66	Male	77

^1^ Non-smoker for 3 years

^2^ Non-smoker for 1 year

^3^ Non-smoker for 11 years before sampling

**Fig. 1:**
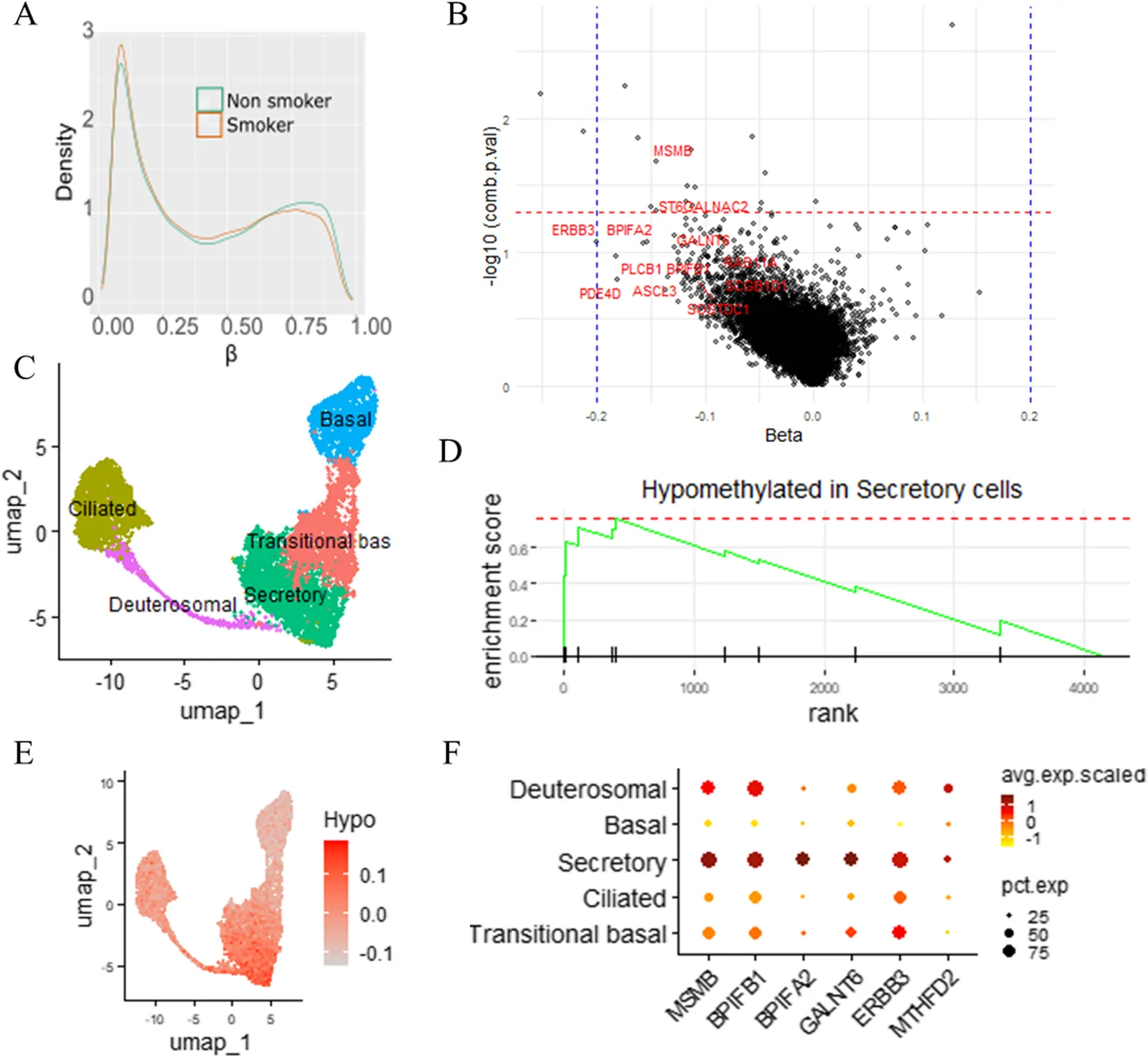
Current smoking-associated promoter hypomethylation in basal cells is linked to secretory cell gene expression. **A** Density plots of promoter β-values in airway basal cells from current smokers and non-smokers. **B** Volcano plot of differential promoter methylation between smokers and non-smokers highlighting leading-edge genes. **C** UMAP of single-cell RNA-seq data from bronchial organoids annotated for major epithelial cells [18]. **D** Gene set enrichment analysis (GSEA) of genes with hypomethylated promoters in secretory cells. **E** UMAP of the organoid epithelium colored by the hypomethylation signature score illustrating preferential enrichment of the hypomethylated-promoter gene set in secretory clusters. **F** Dot plot depicting expression of the leading-edge genes across epithelial cell types

Epigenetic analysis of promoter-associated DNA methylation of basal cells revealed distinct differences between smokers and non-smokers. Density plots of β-values show that current smokers have an increased proportion of hypomethylated promoters compared to non-smokers (Fig. 1A). Across all promoter regions with an absolute methylation difference of at least 5%, 5,115 promoter regions were hypomethylated in smokers, whereas 299 were hypermethylated, indicating a highly significant directional bias toward hypomethylation (exact binomial test, *p* < 2.2 × 10⁻¹⁶). The same pattern was observed when restricting the analysis to nominally significant promoter regions (*p* ≤ 0.05), with 123 hypomethylated and 12 hypermethylated promoter regions in smokers (exact binomial test, *p* = 1.17 × 10⁻²⁴). Together, these data indicate a strong predominance of smoking-associated promoter hypomethylation. The differential methylation analysis was further visualized using a volcano plot (Fig. 1B and S2A). No individual promoter remained significant after correction for multiple testing. Therefore, promoter-level methylation findings are interpreted as exploratory and were used for directionality, pathway-level analyses and integration with transcriptomic data rather than for definitive locus-level inference. To investigate the potential functional relevance of hypomethylated promoters in specific cell types, we performed gene set enrichment analysis (GSEA) using single-cell RNA-seq data generated from our own bronchial organoid model [18], which differentiates into the major airway epithelial cell types (Fig. 1C). The top 100 genes with hypomethylated promoters identified from the epigenetic analysis were tested for enrichment across transcriptional profiles of the different cell clusters. In secretory cells, GSEA revealed a significant enrichment of this gene set (NES = 2.26, *p* = 0.0009) (Fig. 1D). Leading-edge analysis indicated that the enrichment was primarily driven by *MSMB*, *BPIFB1*, *BPIFA2*, *GALNT6*, *ERBB3*, and *MTHFD2* (Fig. 1B). This was further supported by module score analysis, which confirmed that genes with hypomethylated promoters are predominantly upregulated in secretory cell clusters (Fig. 1E, S2B). Consistent with the enrichment results, DotPlot visualization showed that the leading-edge genes were preferentially expressed in secretory cells (Fig. 1F). Notably, the top 100 genes with hypomethylated promoters are also enriched in secretory cells of human airway epithelial tissues (S2C–F [19]), highlighting conservation of their expression pattern between organoids and native tissue. We selected *BPIFB1* for targeted validation because it is a well-established marker of goblet cell differentiation. Targeted bisulfite sequencing of CpG sites within the *BPIFB1* promoter revealed lower methylation in smokers compared with non-smokers (Fig. S2G). Gene ontology (GO) analysis of hypomethylated promoters revealed enrichment in processes related to sensory perception, including detection of chemical stimuli, as well as G protein-coupled receptor signaling and signaling receptor activity (S2H).

**Fig. 2:**
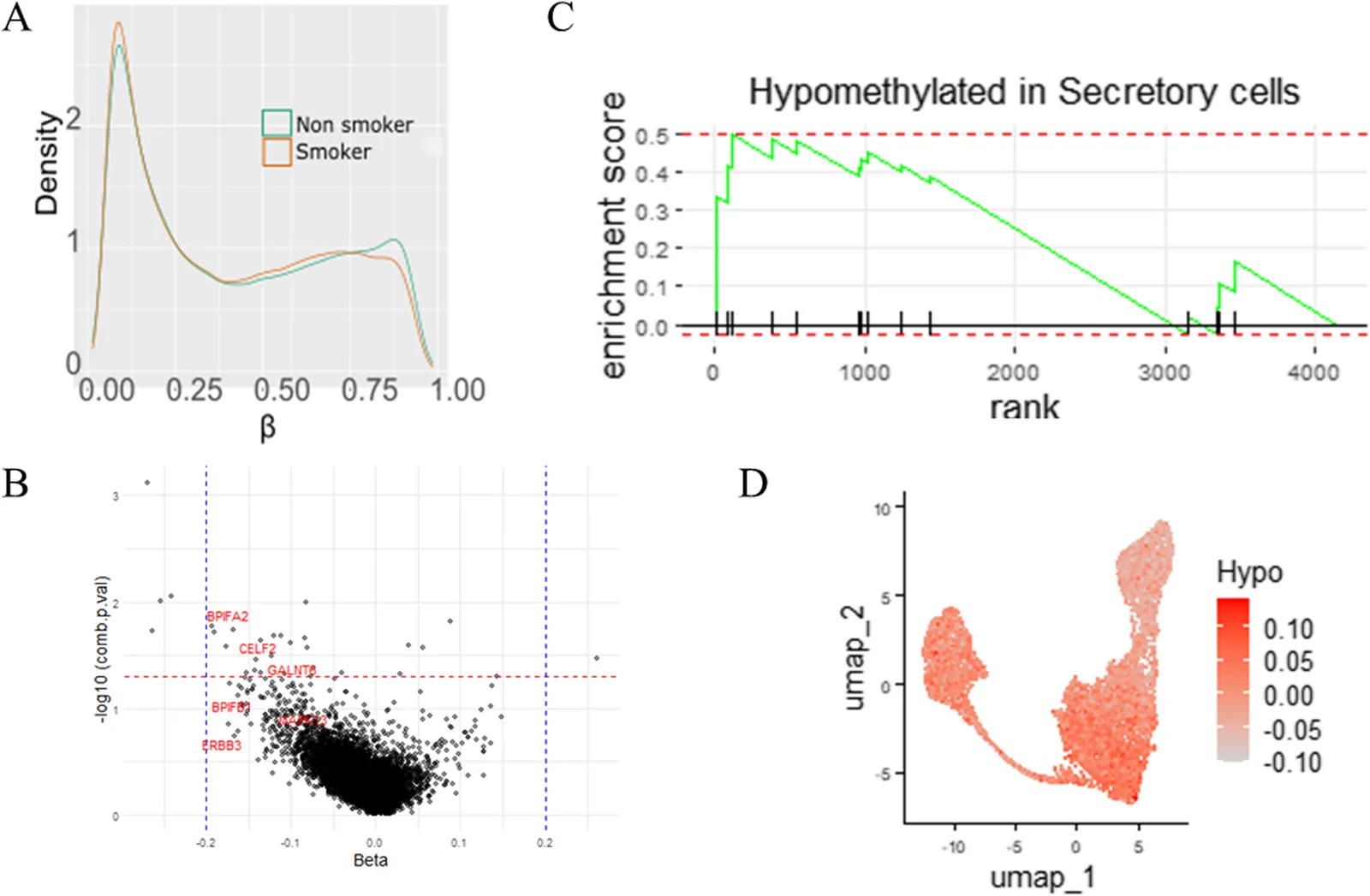
Current smoking-associated promoter hypomethylation in 3D bronchial organoids is linked to secretory cell gene expression. **A** Density plots of promoter β-values in 3D bronchial organoids derived from basal cells of smokers and non-smokers. **B** Volcano plot of differential promoter methylation between smokers and non-smokers highlighting leading-edge genes. **C** Gene set enrichment analysis (GSEA) of genes with hypomethylated promoters in secretory cells within organoids. **D** UMAP of organoid cells colored by the hypomethylation signature score derived from the top 100 hypomethylated-promoter genes illustrating preferential enrichment of this signature in secretory clusters

The basal cells were differentiated to 3D bronchial organoids. Density plots of β-values and volcano plots demonstrated that organoids from smokers exhibited a shift towards promoter hypomethylation (Fig. 2A and B, S3A). Across all promoter regions with an absolute methylation difference of at least 5%, 4,633 promoter regions were hypomethylated in smokers, whereas 1,103 were hypermethylated, indicating a highly significant directional bias toward hypomethylation (exact binomial test, *p* < 2.2 × 10⁻¹4). The same pattern was observed when restricting the analysis to nominally significant promoter regions, with 170 hypomethylated and 20 hypermethylated promoter regions in smokers (exact binomial test, *p* = 3.95 × 10⁻³¹). GSEA of the top 100 genes associated with hypomethylated promoters showed significant enrichment in secretory cells in organoids (NES = 1.72, *p* = 0.0188) (Fig. 2C), driven by leading-edge genes *BPIFB1*, *BPIFA2*, *CELF2*, *GALNT6*, *ERBB3*, and *MAPK13* (Fig. 2B). Module score analysis and dotplot visualization further confirmed that these genes are predominantly expressed in secretory cell clusters, both in organoids and in human airway epithelial tissues (Fig. 2D S3B to F). GO enrichment analysis of genes with hypomethylated promoters revealed enrichment in pathways related to the response to lipopolysaccharide and external stimuli (S3G).

Smoking was also associated with gene-body hypomethylation in basal cells (Fig. S4A, B). Across all gene regions, 3,236 sites were hypomethylated and 222 sites were hypermethylated in smokers. Considering only nominally significant regions, 113 were hypomethylated and 6 were hypermethylated (*p* = 3.51 × 10^⁻27^). GSEA (NES = 1.55, *p* = 0.008) and module score analysis (S4 C to H) revealed a significant enrichment of hypomethylated genes in ciliated cells. The enrichment was primarily driven by genes (e.g., *CFAP126*) exclusively expressed in ciliated cells (S4 I, J). After differentiation of basal cells into organoids 3,086 sites were hypomethylated and 676 sites were hypermethylated in smokers. Considering only statistically significant sites, 117 CpGs were hypomethylated and 11 were hypermethylated (Fig. S5A, B). GSEA did not show significant enrichment of hypomethylated genes in any specific cell type.

These exploratory results indicate that current smoking is associated with increased promoter and gene-body hypomethylation, suggesting systematic deregulation of epithelial differentiation-associated gene programs, particularly in genes expressed in secretory cells.

### Current smoking-associated basal-cell programs emerge as secretory and inflammatory programs during organoid differentiation

**Fig. 3:**
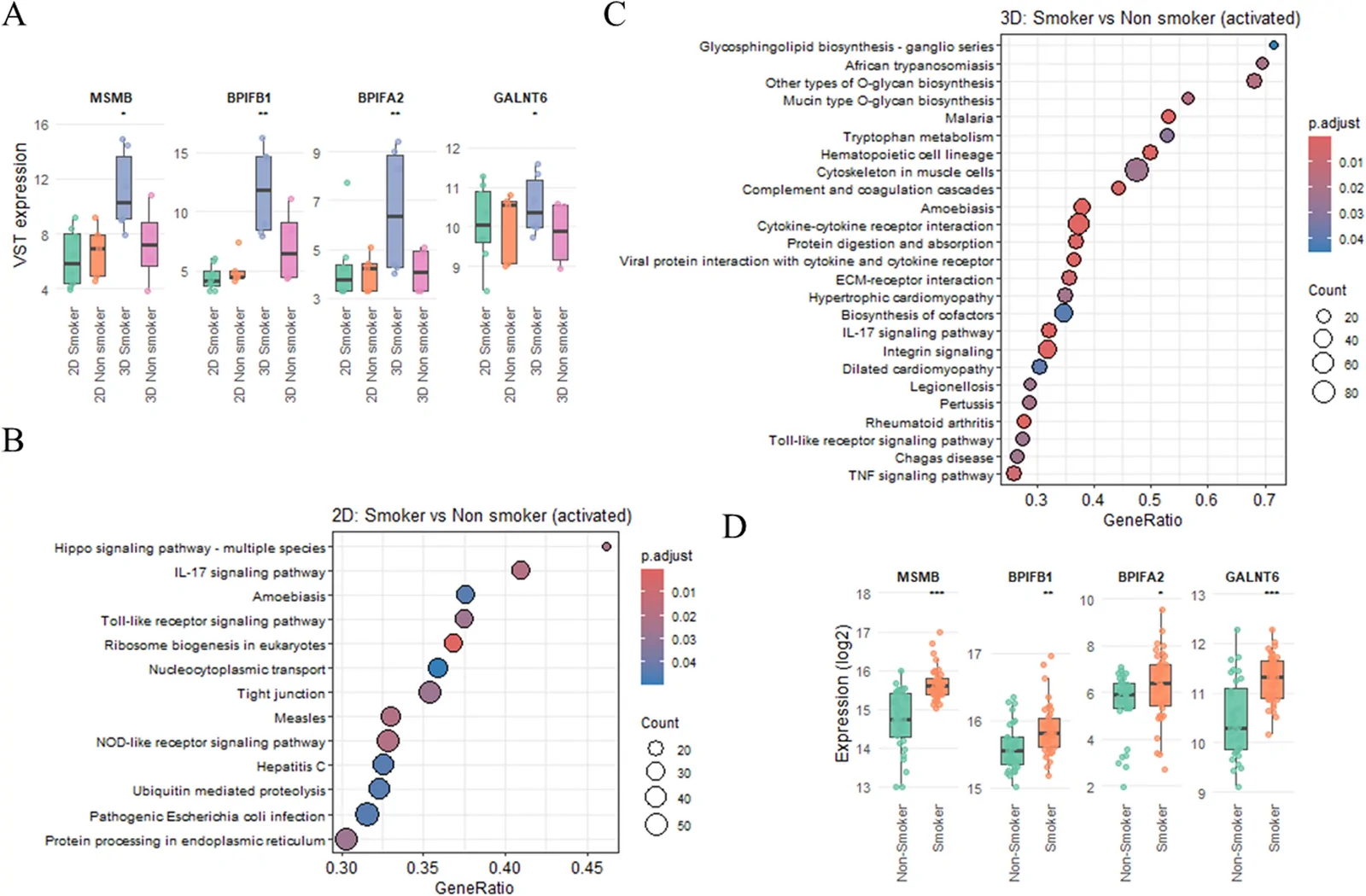
Current smoking is associated with a differentiation-dependent secretory program in basal-cell-derived organoids. **A** Bulk RNA-seq expression of the leading-edge genes in basal cells (2D) and corresponding 3D bronchial organoids from non-smokers and smokers (DESeq2 Wald tests were used for pairwise contrast). **B** KEGG over-representation analysis of genes upregulated in smoker versus non-smoker basal cells. **C** KEGG over-representation analysis of genes upregulated in smoker-derived versus non-smoker 3D organoids. **D** Validation of leading-edge in the airway epithelial brush-biopsy cohort (GSE34450). Log2-normalized expression confirms higher levels in current smokers compared with non-smokers, consistent with the organoid data. Asterisks indicate significance levels from Welch’s t-test (* *p* < 0.05, ** *p* < 0.01, *** *p* < 0.001)

In bulk mRNA-seq of airway basal cells and 3D organoids, the leading-edge genes *MSMB*, *BPIFB1*, *BPIFA2*, and *GALNT6* showed increased expression in organoids (Fig. 3A). Within the 3D condition, samples from smokers displayed higher levels of these genes than those from non-smokers, whereas no significant smoking effect was detected in 2D. While expression levels of additional secretory factors (e.g., *SCGB1A1*, *MUC5AC*) were increased in smokers, other genes expressed in secretory cells, such as *FOXA3* [20], were not associated with smoking status (S6A and B). These findings indicate that smoking-associated transcriptional differences in secretory factors emerge upon epithelial differentiation.

KEGG over-representation analysis of genes upregulated in smokers versus non-smokers in basal cells revealed enrichment of stress- and immune-related pathways, including Hippo signaling, IL-17 signaling, Toll-like and NOD-like receptor signaling, ribosome biogenesis and protein processing in the endoplasmic reticulum (Fig. 3B). In organoids derived from smoker basal cells, an even broader enrichment of inflammatory and host-defense pathways was observed, such as mucin type O-glycan biosynthesis and IL-17 signaling, and multiple infection-related KEGG categories reflecting shared immune, lysosomal, endocytic and cytokine-related gene sets rather than organism-specific exposure (Fig. 3C). KEGG over-representation analysis of genes downregulated in smokers compared with non-smokers in basal cells revealed enrichment of xenobiotic and detoxification pathways, glutathione metabolism and chemical carcinogenesis-DNA adducts (S6C). In organoids derived from smoker basal cells, genes suppressed in smokers were enriched for autophagy-related processes, mRNA surveillance, chromatin remodeling, endocytosis, Notch signalling, nucleotide excision repair and other homeostatic pathways (S6D). These findings suggest that smoking is associated not only with activation of inflammatory programs but also repression of cellular maintenance pathways, particularly after 3D differentiation.

To assess generalizability, we analyzed a public airway epithelial brush-biopsy cohort (GSE34450 [21]). Log2-normalized expression confirmed higher levels of *MSMB*, *BPIFB1*, *BPIFA2*, and *GALNT6* in current smokers compared with non-smokers, consistent with the smoking-associated upregulation observed in our organoid system (Fig. 3D, S6E). The expression of *MSMB* and *BPIFB1* was increased in whole-lung homogenates from current smokers compared with never smokers in the Lung Genomics Research Consortium dataset (GSE47460), whereas *BPIFA2* and *GALNT6* were not represented in this dataset (S6F).

Together, these data show that current smoking is associated with a differentiation-dependent basal-cell program marked by increased secretory and inflammatory gene expression and suppression of homeostatic pathways, consistent with independent airway biopsy data.

### COPD-derived basal cells show methylation-linked activation of host-defense programs

**Fig. 4:**
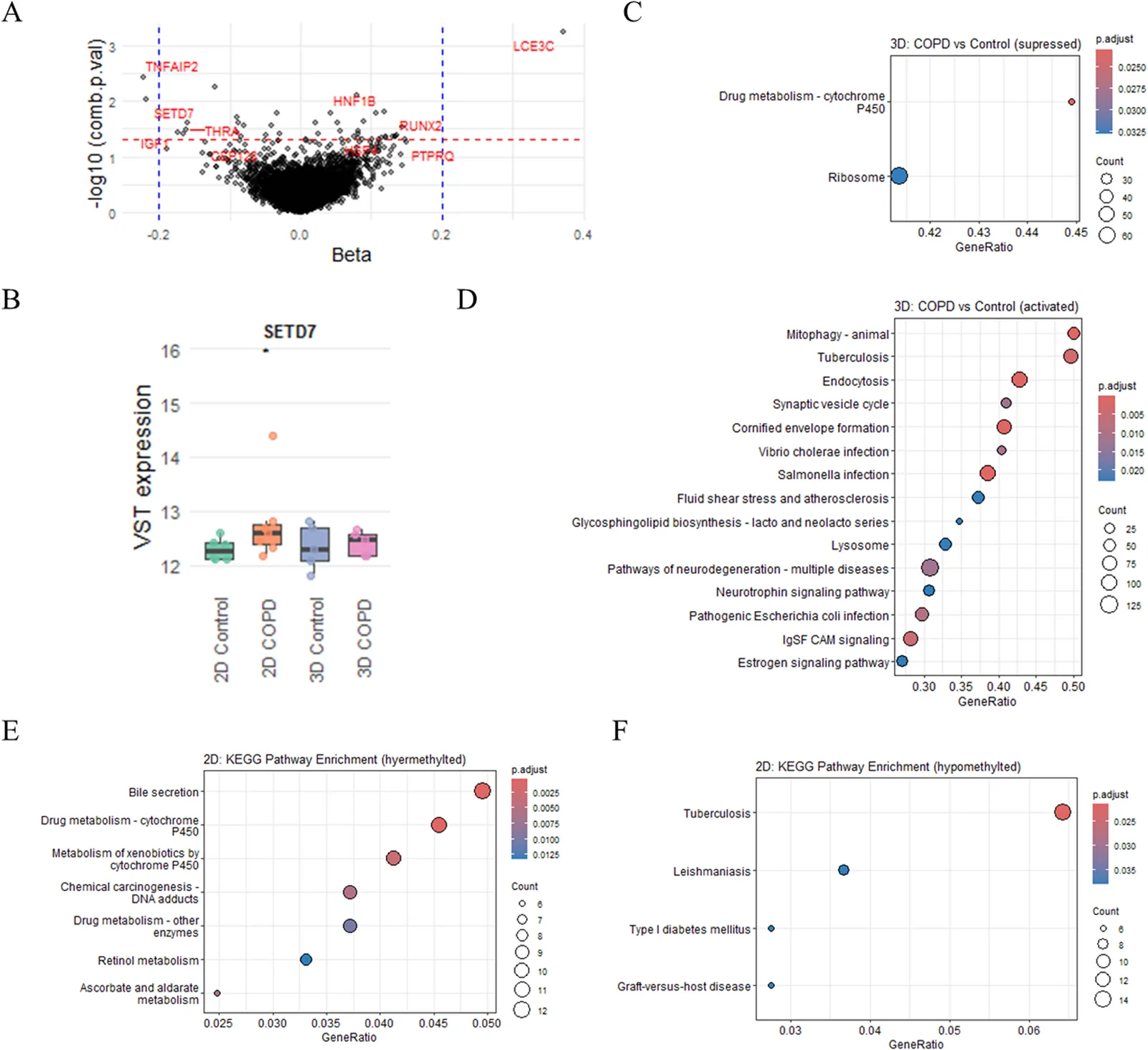
COPD-associated shift from xenobiotic metabolism to infection-related pathways. **A** Volcano plot of promoter-associated methylation in basal cells from COPD patients versus control subjects, highlighting strongly hypomethylated and hypermethylated promoters. **B** Bulk RNA-seq expression of *SETD7*. Asterisks indicate significance levels (* *p* < 0.05, DESeq2 Wald tests were used for pairwise contrast). **C** KEGG over-representation analysis of genes downregulated in COPD organoids. **D** KEGG over-representation analysis of genes upregulated in COPD organoids. **E** KEGG over-representation analysis of the 500 genes with the strongest promoter hypermethylation in COPD basal cells. **F** KEGG over-representation analysis of the 500 genes with the strongest promoter hypomethylation in COPD basal cells

We next assessed epigenetic differences between the COPD patients and control subjects in the study cohort. Density plots of β-values revealed only weak differences in promoter methylation between non-COPD and COPD in either basal cells or organoids (Fig. S7A, S8A). Among the most hypomethylated promoters in COPD patients were those of *TNFAIP2* and *SETD7*. *TNFAIP2* is involved in inflammatory responses [22], while *SETD7* regulates gene expression and inflammation through histone methylation [23]. Conversely, the promoter of *RUNX2*, a transcription factor implicated in cell differentiation [24], was among the ten most hypermethylated promoters (Fig. 4A, S8B). All three genes are expressed in basal and secretory cells (S8C). Bulk mRNA-seq showed that *SETD7* (Fig. 4B), but not *TNFAIP2* (S7B), displayed higher expression in basal cells derived from COPD patients.

KEGG over-representation analysis of genes downregulated in basal cells from COPD patients revealed enrichment of multiple pathways, including ribosome and spliceosome, PI3K-Akt signaling, cytokine-cytokine receptor interaction, complement and coagulation cascades, as well as several infection-related pathways (S7C), whereas genes involved in cornified envelope formation, endocytosis, and autophagy showed increased expression (S7D). KEGG over-representation analysis of genes downregulated in 3D airway organoids from COPD patients revealed drug metabolism-cytochrome P450 pathways and enrichment of ribosome (Fig. 4C). Genes upregulated in COPD organoids were enriched for multiple pathways, including sphingolipid signaling, several infection-related pathways such as tuberculosis and pathogenic *Escherichia coli* infection, lysosome, and endocytosis (Fig. 4D). In addition, KEGG pathway analysis was performed on the 500 genes with the strongest promoter methylation changes in basal cells, ranked by combined *p*-value and classified according to the direction of change (hypermethylation or hypomethylation). Genes with hypermethylated promoters were enriched for pathways related to xenobiotic metabolism, including drug metabolism–cytochrome P450 (Fig. 4E), whereas genes with hypomethylated promoters showed enrichment of immune- and infection-related pathways, including tuberculosis and graft-versus-host disease (Fig. 4F). GO analysis revealed that hypomethylated promoters in basal cells were enriched for pathways related to cell activation, acute phase response, and lysosome and endosome organization (Fig. S7E).

Together, these data indicate that basal cells in COPD exhibit promoter hypermethylation and reduced activity of xenobiotic metabolism pathways, concomitant with promoter hypomethylation and increased activation of infection- and inflammation-related pathways, which becomes particularly evident in differentiated 3D organoids.

## Discussion

In this study, we provide exploratory evidence that current smoking and COPD are associated with distinct yet converging methylation-linked and transcriptional imprints in human airway basal cells that become functionally apparent upon epithelial differentiation. Current smoking was associated with widespread promoter hypomethylation in basal cells and derived 3D bronchial organoids, with hypomethylation preferentially affecting genes that are highly expressed in secretory cells. While these changes were already detectable at the epigenetic level in undifferentiated basal cells, current smoking-associated upregulation of secretory and inflammatory genes emerged predominantly after 3D differentiation and was accompanied by repression of homeostatic pathways. Analysis of a publicly available airway brush-biopsy cohort confirmed increased expression of key hypomethylated secretory genes in current smokers. In COPD, basal cells showed promoter hypermethylation of xenobiotic metabolism genes alongside promoter hypomethylation of immune- and infection-related genes. Consistent with this shift, COPD-derived organoids displayed attenuated xenobiotic metabolism and enhanced lysosomal, endocytic and immune-related pathways. Together, these data support a model in which current smoking and COPD are linked to basal-cell programs that bias epithelial progeny away from epithelial homeostasis towards secretory, inflammatory and infection-related gene expression.

A key finding is that current smoking is associated with global hypomethylation in airway basal cells and their derived organoids. The most prominently hypomethylated promoters in basal cells are enriched for genes preferentially expressed in secretory cells, including *BPIFB1*, *BPIFA2*,* MSMB*, and *GALNT6*. Integration with organoid single-cell RNA-seq and independent human airway datasets [19] shows that these genes are consistently and robustly expressed in secretory cells in both organoids and native tissue. Together, these observations point to a targeted reconfiguration of regulatory regions linked to secretory differentiation in the airway epithelium of smokers. A second important observation is the differentiation dependence of the smoking-associated transcriptional response. Despite hypomethylation at secretory gene promoters in basal cells, these genes show low expression and no smoking-associated differences in 2D cultures. After 3D differentiation, however, organoids derived from smoker basal cells markedly upregulate these genes, and this pattern is recapitulated in independent airway biopsy data [21]. These findings support a model in which basal cells retain an epigenetic memory of smoking that remains latent in undifferentiated culture but becomes apparent during differentiation. This concept aligns with the idea that basal cells are long-lived progenitors that store environmental information and pass it on to their epithelial progeny [25–28]. Consistent with our findings, Buro-Auriemma et al. [12] reported a pronounced predominance of smoking-associated hypomethylation (approximately threefold more hypo- than hypermethylated loci) in a genome-wide methylation analysis of human small airway epithelium (bronchial brushing), with changes typically focal and frequently located within 2 kb of the transcription start site. Notably, hypomethylation was enriched among xenobiotic metabolism and detoxification genes (e.g., *CYP1A1*, *CYP1B1*, *ALDH3A*), which have been shown to be preferentially expressed in secretory airway epithelial cells [18, 29], supporting the concept of smoke-induced epigenetic priming of epithelial stress-response programs in secretory cells [12]. Buro-Auriemma et al. [12] reported hypomethylation of xenobiotic metabolism and detoxification genes in directly sampled small airway epithelium from smokers, whereas our ex vivo basal-cell and organoid data point to reduced detoxification-associated transcriptional programs, most prominently in COPD-derived organoids. This apparent discrepancy may be explained by differences in the sampled epithelial compartment and exposure context, as native airway brushings capture directly smoke-exposed epithelium in vivo, while our model assesses expanded basal cells and smoke-free organoid differentiation. Thus, acute smoke-induced xenobiotic metabolism responses may no longer be present in our culture system, whereas persistent progenitor-associated or disease-associated reductions in detoxification-related homeostatic capacity may become more apparent.

In basal cells, genes upregulated in smokers showed pathway enrichment for Hippo, IL-17, Toll-like and NOD-like receptor signaling, as well as for pathways involved in ribosome biogenesis and endoplasmic reticulum protein processing, suggesting that smoking reshapes key regulatory and stress-response networks in airway progenitors [30, 31]. It has been shown that YAP/TAZ, which are negatively regulated by the upstream hippo kinase cascade, maintain airway epithelial architecture by preserving basal stem cell identity and restraining aberrant goblet cell differentiation, thereby supporting lung epithelial homeostasis [32, 33]. While enrichment of Hippo pathway genes in basal cells obtained from smokers does not allow us to infer the direction of YAP/TAZ activity, it suggests that smoking affects key stem cell and lineage-control pathways upstream of differentiation. Consistent with an expansion of secretory and host-defense programs, genes upregulated in smoker-derived organoids were enriched for mucin-type O-glycan biosynthesis, IL-17 signaling and multiple infection-related KEGG pathways, indicative of an intrinsically hypersecretory and inflammation-primed epithelial state. IL-17 has been shown to induce MUC5AC expression and goblet cell hyperplasia, supporting a mechanistic link between IL-17-dominated signaling and the hypersecretory, inflammation-primed state observed in smoker-derived organoids [34, 35] In parallel, genes downregulated in smokers showed enrichment for autophagy, mRNA surveillance, chromatin remodeling and endocytosis pathways in organoids, pointing to a concomitant attenuation of epithelial detoxification and cellular maintenance mechanisms. These findings are consistent with reports of autophagy insufficiency and disturbed proteostasis in bronchial epithelial cells and COPD lungs from smokers, and support the hypothesis that impaired autophagy contributes to smoke-induced remodeling of the airways [36–38]. Together, these data support the concept that smoking reprograms basal cells into a progenitor state that is more prone to generating mucus-producing, inflammation-responsive epithelial subsets with altered homeostatic capacity upon differentiation. This notion is consistent with recent work by Carlier et al. [26], who showed that bronchial epithelial air–liquid interface cultures derived from current smokers, even when maintained for weeks, retain persistent defects in barrier function and lineage allocation, including increased *SPDEF* and *MUC5AC* expression, indicating an intrinsic, long-lasting imprint of cigarette smoke on the airway epithelium.

Our data also reveal a coordinated epigenetic and transcriptional reprogramming in airway basal cells from COPD patients that becomes functionally visible upon epithelial differentiation. Although global promoter methylation patterns were similar between COPD and control subjects, specific loci showed pronounced shifts. Promoter hypomethylation of *TNFAIP2* and *SETD7*, together with hypermethylation of the differentiation-associated transcription factor *RUNX2*, indicates that COPD basal cells acquire a regulatory landscape skewed towards inflammatory priming and away from homeostatic differentiation programs. Consistent with this, *SETD7* expression was elevated in COPD-derived basal cells, supporting its role as an epigenetic mediator of inflammatory and stress-related gene regulation in chronic airway disease.

Strikingly, the pathway-level consequences of methylation changes in basal cells are mirrored in the transcriptomes of their 3D-derived organoids. KEGG analysis of hypermethylated promoters revealed enrichment for xenobiotic metabolism and cytochrome P450 pathways essential for detoxification of inhaled toxicants. Correspondingly, 3D organoids from COPD patients showed transcriptional downregulation of xenobiotic metabolism-related pathways, including cytochrome P450 pathways. This suggests that epigenetic repression of detoxification capacity is already established in basal progenitors and maintained during differentiation. In contrast, hypomethylated promoters in basal cells derived from COPD-patients were enriched for infection- and immunity-related KEGG categories such as tuberculosis, leishmaniasis, type I diabetes, and graft-versus-host disease, along with GO terms related to cell activation, acute-phase responses, and lysosome and endosome organization. This epigenetic priming is recapitulated in 3D organoids, where infection-related pathways, including tuberculosis, Gram-negative pathogenic bacteria (*Escherichia coli* infection, *Salmonella*, *Vibrio*), as well as lysosome and endocytosis pathways were among the most strongly upregulated.

Together, these findings indicate that basal stem cells from COPD patients harbor methylation-linked regulatory programs that biases their differentiated progeny towards heightened inflammatory programs at the expense of detoxification and metabolic homeostasis. This concept is consistent with studies showing that airway epithelial cells from COPD patients exhibit cell-intrinsic, long-lasting abnormalities in barrier function, polarity, ciliogenesis and lineage allocation when cultured in air-liquid interface or 3D scaffold models under smoke-free conditions, indicating a persistent epithelial memory of COPD-related injury [26, 39–41]. Our combined methylome-transcriptome data extend these observations by placing such durable shifts upstream in the basal stem-cell compartment and by implicating differential promoter methylation of detoxification and infection-related genes as a potential mechanism encoding this epithelial memory. This shift aligns with clinical features of COPD. Reduced xenobiotic metabolism may decrease the epithelial capacity to neutralize environmental toxicants, thereby increasing susceptibility to oxidative injury. Concurrent activation of infection- and inflammation-related as well as lysosomal and endocytic activation pathways likely reflects chronic microbial exposure and inflammation, impaired mucociliary clearance, and repeated airway injury characteristic of COPD [42].

Our work also highlights both the strengths and limitations of patient-derived airway organoids for dissecting human epithelial pathobiology. The use of matched basal cells and 3D organoids from the same individuals allowed us to demonstrate that smoking- and COPD-associated methylation changes established in basal cells translate into distinct transcriptional states only after differentiation, underscoring the importance of epithelial architecture and microenvironmental cues for disease-relevant gene regulation. The integration of bulk methylation, bulk RNA-seq, single-cell RNA-seq and independent airway biopsy data strengthens the biological coherence of our findings and reduces the likelihood that they reflect model-specific artefacts. However, this study is limited by the modest cohort size and the clinical context of control donor recruitment, as controls were obtained in a lung cancer surgery setting and may therefore not fully represent a healthy reference population. Although only non-malignant airway-derived material was analyzed, residual effects of donor heterogeneity, clinical background, and confounding by sex, cumulative smoke exposure, medication, and comorbidities cannot be fully excluded. Our findings should therefore be interpreted as biologically consistent patterns that support the underlying hypothesis within this cohort, rather than as definitive evidence.

A central limitation is that the primary smoking comparison was not based on a uniform never-smoker control group. Instead, current smokers were compared with current non-smokers, including both never smokers and former smokers. This distinction is important because smoking-associated molecular alterations do not uniformly disappear after cessation [7, 43, 44]. Airway epithelial gene-expression studies have identified both reversible and persistent smoking-associated signatures in former smokers [43, 45]. DNA methylation studies indicate that recovery after smoking cessation is incomplete, locus-dependent and may occur over variable time scales [7, 44]. While many smoking-associated DNA methylation changes decline after cessation, some CpG sites can remain altered for years [7, 44]. Accordingly, residual smoking-related methylation signatures in former smokers cannot be excluded and may have attenuated group differences. This limits a strict interpretation of our findings as a never-smoker versus current-smoker comparison and supports their interpretation as exploratory, current-smoking-associated epithelial programs within this cohort. A further limitation is that no promoter remained significant after multiple-testing correction in the smoker versus non-smoker comparison. Thus, the promoter-level methylation findings should be considered exploratory rather than definitive. Nevertheless, these findings were embedded in a consistent global bias toward promoter hypomethylation in smokers and were concordant with RNA-seq changes, supported by targeted *BPIFB1* validation, and reinforced by external datasets, indicating a biologically coherent smoking-associated epithelial program across orthogonal data layers. In addition, the methylation data are limited to CpG sites represented on the array platform, and the cross-sectional design prevents firm conclusions about causality or reversibility.

Organoid models provide a standardized differentiation context, but they may also introduce culture-associated epigenetic and transcriptional changes that should be considered when interpreting disease-associated findings. In intestinal epithelial organoids, prolonged culture has been associated with reproducible DNA methylation changes, including global hypomethylation, increased variability, and partial effects on gene expression and cellular function [46]. In addition, while organoids can retain key epigenetic features of the tissue of origin, developmental or maturation-associated methylation changes have also been described [47]. Consistent with this general principle, even technical processing of primary airway epithelial cells can measurably affect DNA methylation profiles [48]. Thus, the smoking- and COPD-associated programs that became most apparent after 3D differentiation in our study may reflect not only pre-existing epithelial imprinting, but also its manifestation or accentuation within the organoid context. Nevertheless, because all groups were processed under identical culture conditions and basal-cell methylation and transcriptomic profiling were generated from matched starting material prior to organoid culture, organoid development alone is unlikely to explain the observed group-specific differences. More generally, organoids remain a reductionist system lacking immune cells, vascular components, mechanical forces, and the resident microbiome, all of which shape epithelial behavior in vivo.

In summary, our data support the presence of smoking- and COPD-associated methylation-linked epithelial programs in human airway basal cells that nevertheless converge during differentiation on a shared functional outcome: a shift away from epithelial homeostasis toward secretory, inflammatory and infection-related gene programs. The concordance between promoter methylation changes in basal cells and pathway-level transcriptional states in matched 3D organoids supports the concept of a durable epithelial memory encoded in the stem-cell compartment that becomes apparent after differentiation. Despite the modest cohort size and the reductionist nature of organoids, these findings provide a mechanistic framework to explain persistent, cell-intrinsic epithelial remodeling in smokers and COPD. Future studies should test the causal contribution of key regulatory loci and assess whether these epigenetic imprints are therapeutically reversible.

## Methods

### Organoid culture

Primary human bronchial epithelial cells (HBECs) were isolated from bronchial brushings of 13 donors (Table 1) during routine examinations and cultured on 3T3 feeder layers, as previously described [18, 49, 50]. Cells were differentiated into bronchospheres, as previously described [18, 51]. The study protocol was approved by the Ethics Committee (Institutional Review Board) of the Saarland State Medical Association, and informed consent was obtained from all patients. Briefly, 40 µL of a 25% Matrigel solution (Corning, USA) in differentiation medium (DM; Lonza, Switzerland), mixed 1:1 with DMEM/F12 (Gibco, USA) containing 50 nM retinoic acid, was added to each well of a 96-well plate. Subsequently, 80 µL of HBECs (3 × 10⁴ cells/mL, passages 1–2) in DM supplemented with 5% Matrigel and 50 nM retinoic acid were added to each well. Total DNA and mRNA were extracted from basal cells on the day of seeding and after a differentiation period of 21 days.

### Epigenetic analysis

Detailed downstream analyses are described in the Supplementary Methods. For each sample, 700 ng of genomic DNA was bisulfite converted using the EZ DNA Methylation-Gold Kit (#D5006, Zymo Research, USA) according to the manufacturer’s instructions, with a final elution volume of 10 µl. DNA methylation was profiled using the Infinium MethylationEPIC v2.0 BeadChip (Illumina, USA) and scanned on an Illumina iScan platform. Raw data were processed with RnBeads (v2.26.0) and mapped to the human reference genome hg38 (GRCh38). Quality control included inspection of beta-value distributions, coverage histograms, boxplots, and SNP-based checks. Probes on sex chromosomes, low-coverage sites (< 5×), CpGs in non-CpG contexts, and SNP-overlapping sites (minor allele frequency > 3%) were excluded. Additional filtering of unreliable probes was performed using the Greedycut algorithm with a detection *P*-value threshold of ≥ 0.05. In total, 59,081 probes were removed, while all samples passed quality control. Data were normalized using BMIQ without background correction. Differential methylation analysis between groups was performed in RnBeads using limma at the CpG-site level. For promoter and gene regions, methylation values were aggregated across CpGs and summarized using the RnBeads combined *p*-value statistic (comb.p.val). Output tables for promoters and genes were exported for downstream analysis in R. Promoters were ranked by comb.p.val, and group-wise mean methylation differences (Δβ; mean.mean.diff) were extracted. Gene-level annotation was added using org.Hs.eg.db and AnnotationDbi, and analyses were restricted to protein-coding genes. Promoters were classified as hypomethylated (Δβ < 0) or hypermethylated (Δβ > 0), ranked by the combined RnBeads score, and visualized by volcano plot. For pathway analysis, the top 500 hypo- and hypermethylated promoters were analyzed separately by KEGG over-representation analysis using clusterProfiler::enrichKEGG (organism = “hsa”, *p*-value cutoff = 0.05) after mapping gene symbols to Entrez IDs. To assess whether methylation-defined gene sets were enriched in specific epithelial cell populations, we integrated the epigenetic results with our single-cell RNA-seq data from organoids and native proximal airway tissue.

### Bulk RNA sequencing

Further details on external dataset processing and downstream enrichment analyses are provided in the Supplementary Methods. Total RNA concentration and purity were assessed using a NanoDrop 8000 spectrophotometer (Thermo Fisher Scientific, USA). Libraries for bulk mRNA sequencing were prepared and sequenced on a DNBSeq PE150 platform (BGI Tech Solutions) with 20 million paired-end reads per sample. Reads were aligned to the human reference genome GRCh38 using STAR (v2.7.3a), and gene-level count tables were generated with featureCounts (v2.0.1). Count matrices from two sequencing runs were processed in R using DESeq2. Ensembl gene identifiers were cleaned and mapped to HGNC symbols using biomaRt, genes without unique mappings were removed, and both runs were harmonized by intersecting shared genes and merging the count matrices and metadata. Experimental groups were encoded as a four-level factor reflecting smoking status and culture condition (Yes_2D, No_2D, Yes_3D, No_3D). After filtering lowly expressed genes, differential expression analysis was performed in DESeq2 using negative binomial generalized linear models with Wald tests, and log2 fold changes were shrunken with apeglm. *P* values were adjusted across genes using the Benjamini–Hochberg procedure. KEGG over-representation analysis was performed with clusterProfiler using significantly regulated genes (padj ≤ 0.05 and absolute shrunken log2 fold change ≥ 0.58) after mapping HGNC symbols to Entrez identifiers; the background was defined as all genes with valid Entrez IDs in the DESeq2 results. Published transcriptomic datasets from GSE34450 and GSE47460 were obtained from GEO and processed in a harmonized manner. Smoking status and disease annotations were extracted from sample metadata, relevant samples were retained according to predefined inclusion criteria, probe sets were mapped to HGNC gene symbols, and multiple probes per gene were summarized by the mean. For predefined candidate genes, expression differences between smoking groups were assessed using two-sided Welch’s t-tests and visualized by boxplots.

## Supplementary Information

Supplementary Material 1.

Supplementary Material 2.

## Data Availability

The datasets generated during the current study are available under accession number GSE326391. Publicly available datasets analyzed in this study are available under accession numbers GSE34450 and GSE47460. Additional processed data are included in this article and its supplementary information files or are available from the corresponding author on reasonable request.
