# Supplementary material for: Current smoking and COPD are associated with differentiation-dependent secretory and inflammatory programs in airway basal cells
Source: Respir Res. 2026 Aug 29;27:343. doi: 10.1186/s12931-026-03847-4 (PMC13543530; doi:10.1186/s12931-026-03847-4)
Supplement: Supplementary file 1 [file 12931_2026_3847_MOESM1_ESM.pdf]

Current smoking and COPD are associated with differentiation-dependent secretory and inflammatory programs in airway basal cells

Felix Ritzmann, Michelle Brand, Gilles Gasparoni, Xuan Zhang, Frank Langer, Christian Herr, Yiwen Yao, Migdat Mustafi, Daniela Yildiz, Jörn Walter, Robert Bals, Christoph Beisswenger

ONLINE DATA SUPPLEMENT

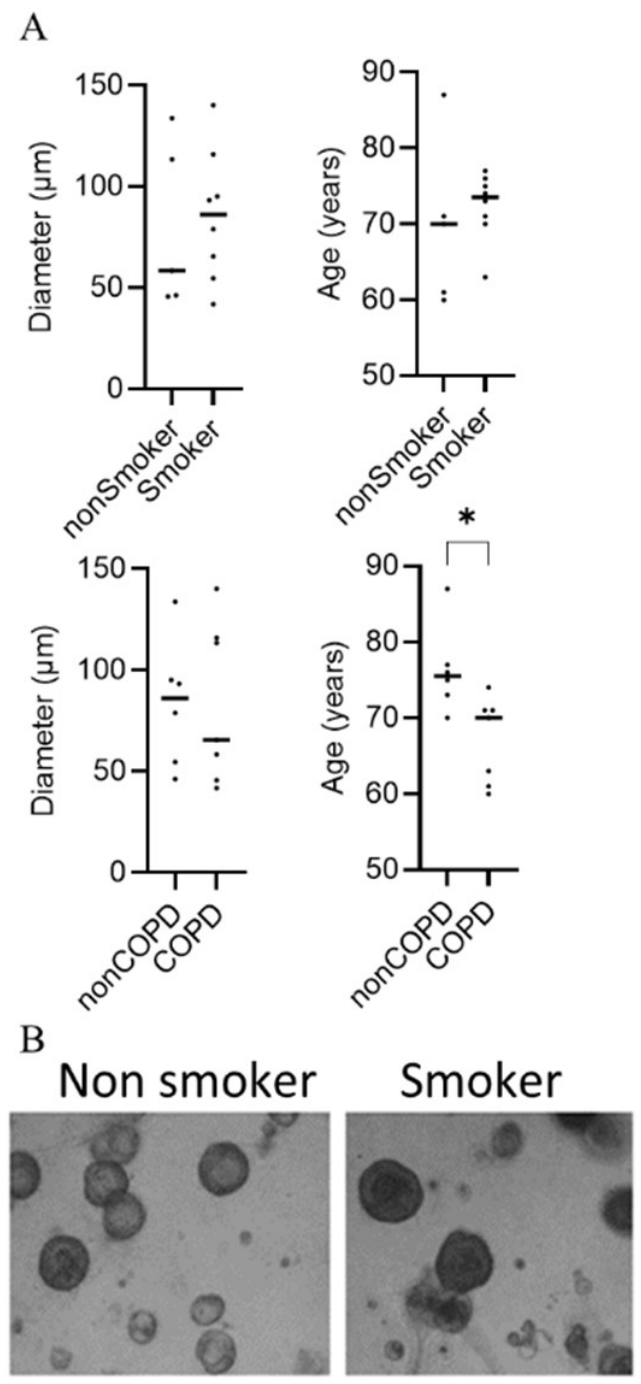

S1. (A) organoid diameters and age of donors. (B) Representative phase-contrast images of organoids.

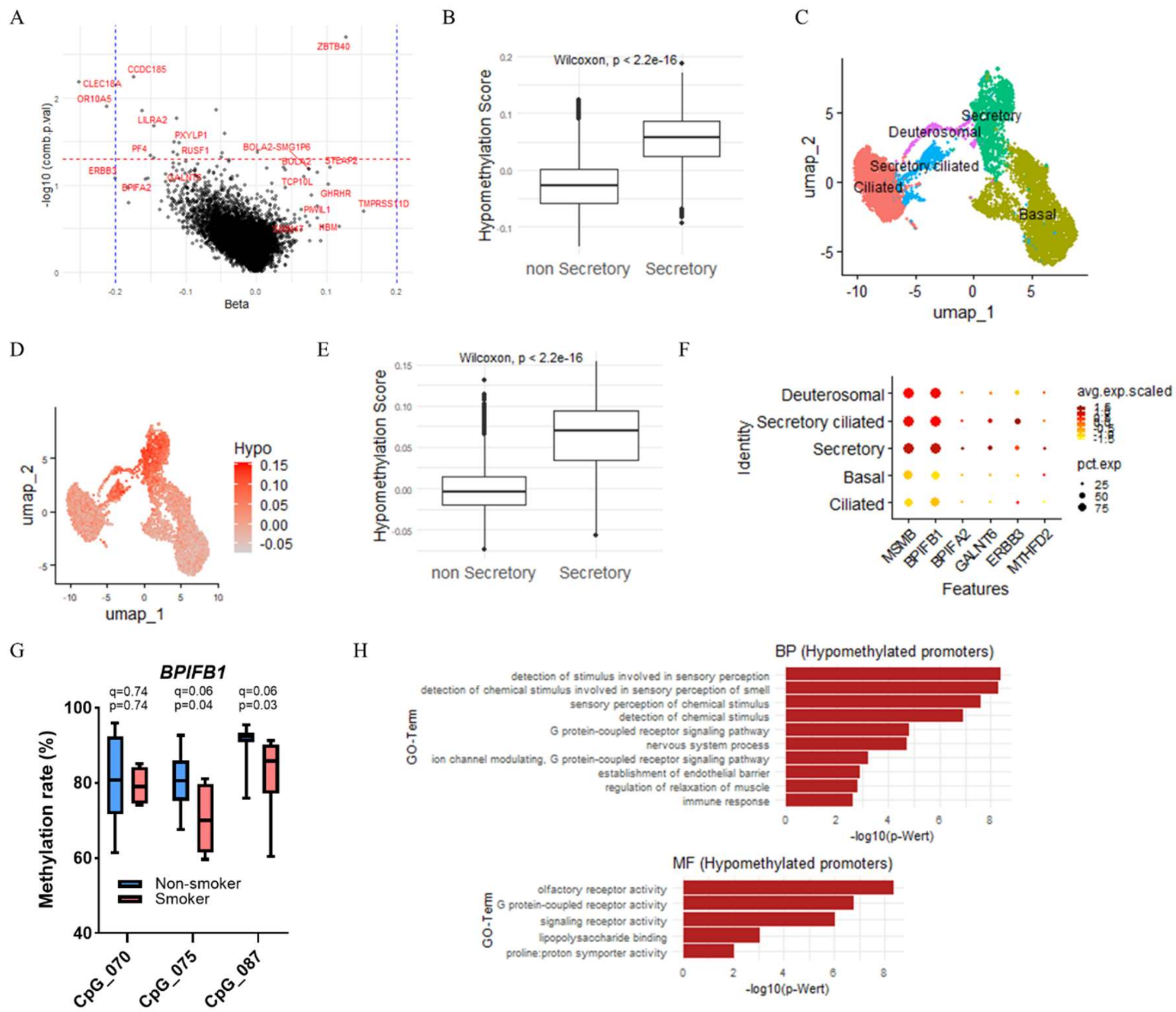

S2. Current smoking-associated promoter hypomethylation in basal cells is linked to secretory cell gene expression. (A) Volcano plot of differential promoter methylation between smokers and non-smokers. (B) Boxplot of hypomethylation signature scores (based on the top 100 hypomethylated promoters) comparing non-secretory versus secretory epithelial cells. (C) UMAP of an independent airway epithelial dataset (C to F) from the proximal lung [19] annotated for major epithelial cell types. (D) UMAP colored by the hypomethylation signature score indicating preferential enrichment of the hypomethylated-promoter gene set in secretory and secretory-ciliated clusters. (E) Boxplot of hypomethylation signature scores demonstrating higher scores in secretory compared with non-secretory cells. (F) Dot plot showing expression of the leading-edge genes. (G) CpG methylation was quantified by bisulfite conversion followed by targeted amplicon sequencing. Bars indicate the median and whiskers indicate the range. Group differences were tested for each CpG using two-sided Mann–Whitney U tests; nominal p values are shown. P values were adjusted for multiple comparisons across the three CpGs using the Benjamini–Hochberg false discovery rate (FDR); FDR-adjusted q values are shown. Smoker n=7, Non-Smoker n=6. (H) Gene ontology (GO) for biological process (BP) and molecular function (MF) enrichment for genes with hypomethylated promoters.

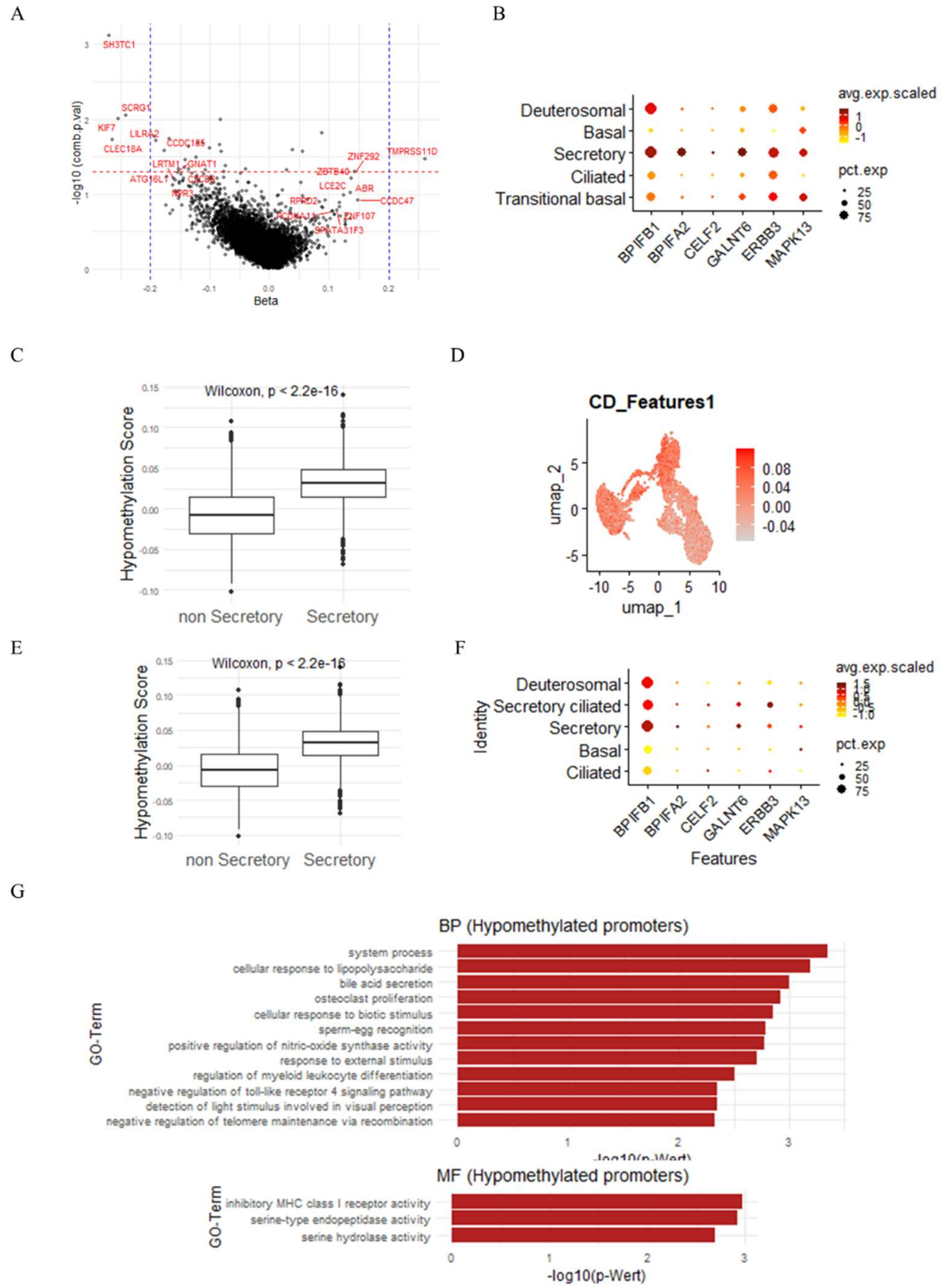

S3. Current smoking-associated promoter hypomethylation in 3D bronchial organoids is linked to secretory cell gene expression. (A) Volcano plot of differential promoter methylation in 3D organoids. (B) Dot plot showing expression of the leading-edge genes. (C) Boxplot of hypomethylation signature scores comparing non-secretory and secretory epithelial cells in organoids. (D) UMAP of human airway epithelial single-cell data colored by the hypomethylation signature score illustrating enrichment of the organoid-derived hypomethylation gene set in secretory and secretory-ciliated clusters in native tissue (D to F). (E) Boxplot of hypomethylation signature scores in the airway epithelial tissue dataset comparing non-secretory and secretory cells. (F) Dot plot showing expression of the leading-edge genes in airway tissue dataset. (G) GO for genes with hypomethylated promoters in 3D organoids.

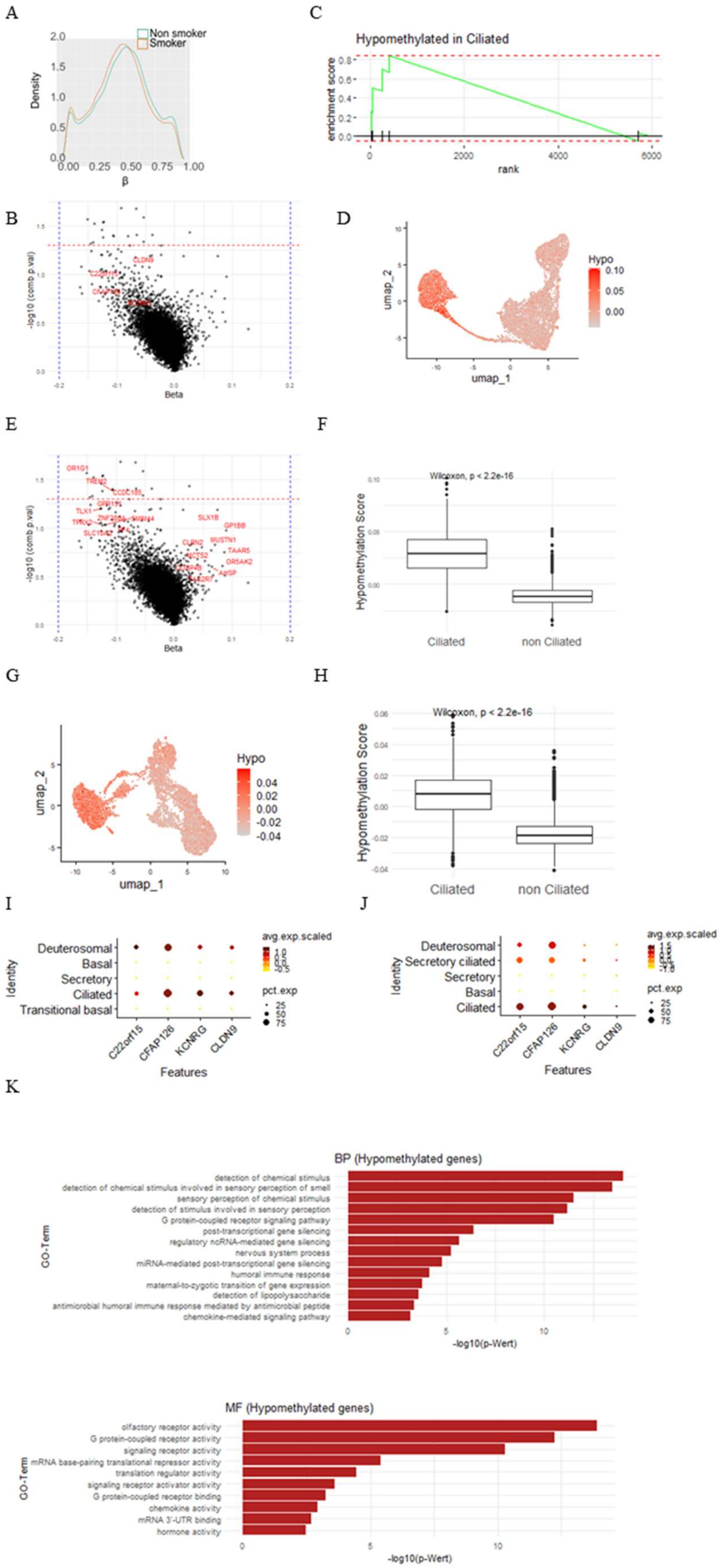

S4. Current smoking-associated gene-body hypomethylation in basal cells is linked to ciliated cells. A) Density plots of β-values for CpGs sites across gene bodies in basal cells from smokers and nonsmokers

highlighting leading-edge genes. (B) Volcano plot of differential gene-body methylation between smokers and non-smokers highlighting leading-edge genes. (C) Gene set enrichment analysis (GSEA) of hypomethylated genes in ciliated cells. (D) UMAP of organoid cells colored by the gene-body hypomethylation signature score derived from the top 100 genes illustrating preferential enrichment of this signature in ciliated cell clusters. (E) Volcano plot of differential gene-body methylation between smokers and non-smokers. (F) Boxplot of gene-body hypomethylation signature scores comparing ciliated and non-ciliated epithelial cells in the organoid single-cell dataset showing higher scores in ciliated cells. (G) UMAP of airway epithelial cells (independent human airway epithelial dataset) colored by the hypomethylation signature score illustrating localization of the signature to ciliated regions of the epithelium. (H) Boxplot of hypomethylation signature scores showing enrichment of the signature in ciliated cells. (I) Dot plot showing expression of leading-edge genes in the organoid dataset. (J) Dot plot of leading-edge genes in the independent airway tissue dataset. (K) GO for genes with hypomethylated promoters.

A

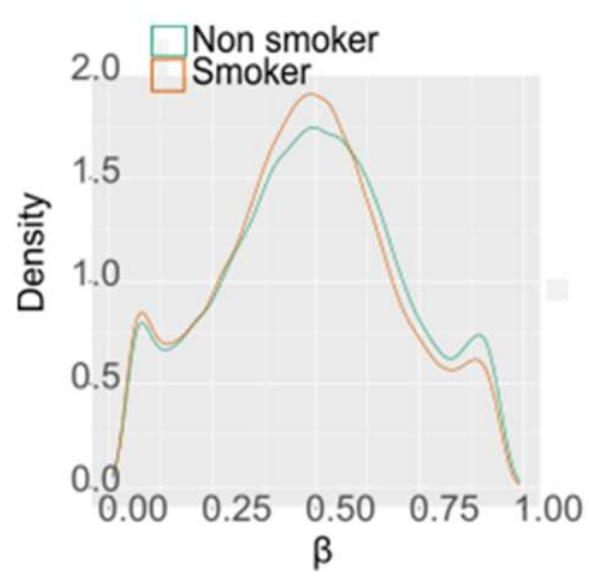

B

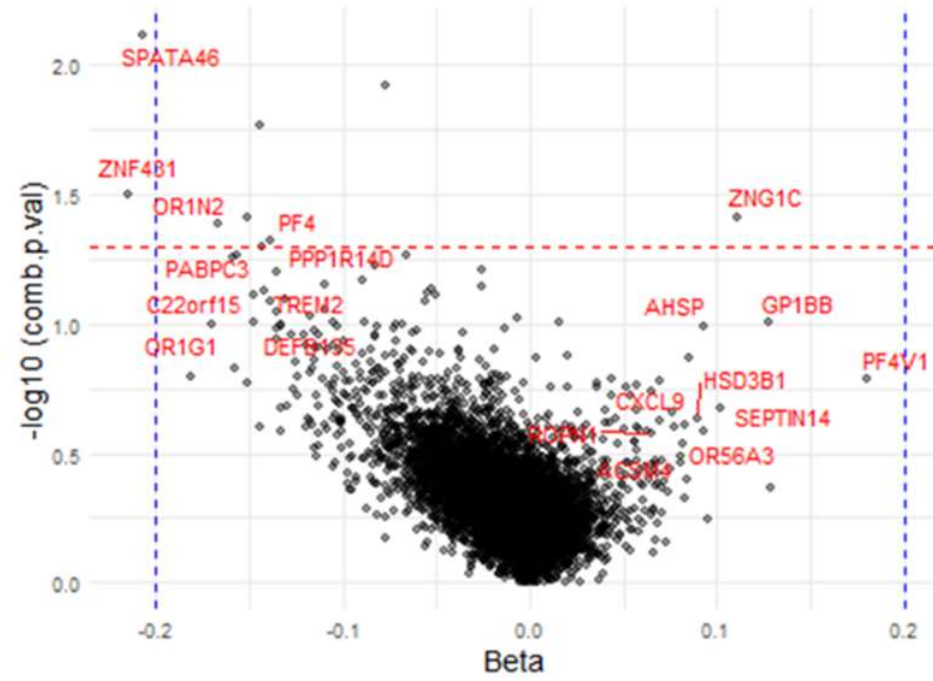

C

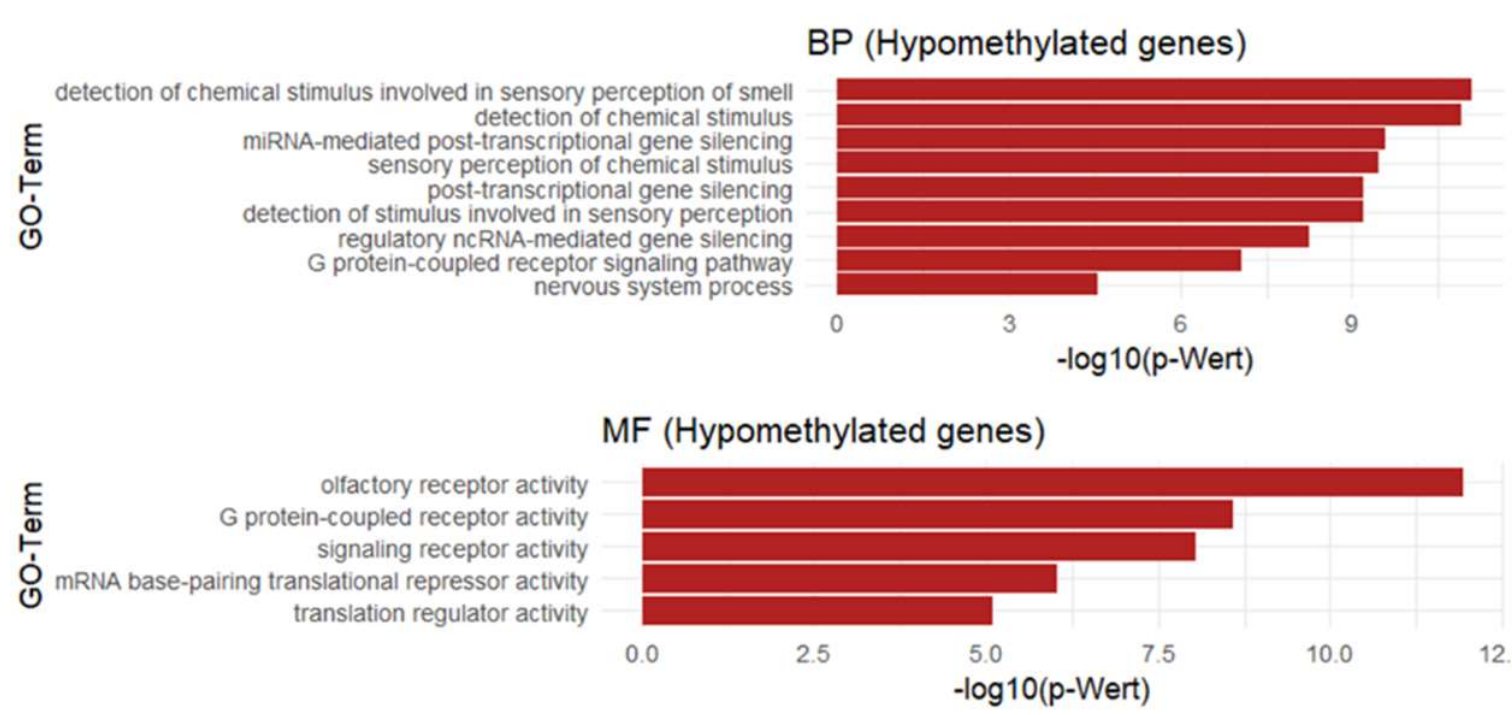

S5. Smoking-associated gene-body hypomethylation in 3D bronchial organoids. (A) Density plots of gene-body β-values in 3D bronchial organoids derived from basal cells of smokers and non-smokers. (B) Volcano plot of differential gene-body methylation between smokers and non-smokers. (C) GO biological process enrichment analysis for genes with hypomethylated gene bodies in organoids, showing over-representation of pathways related to detection of chemical stimuli and sensory perception. (D) GO for genes with hypomethylated promoters.

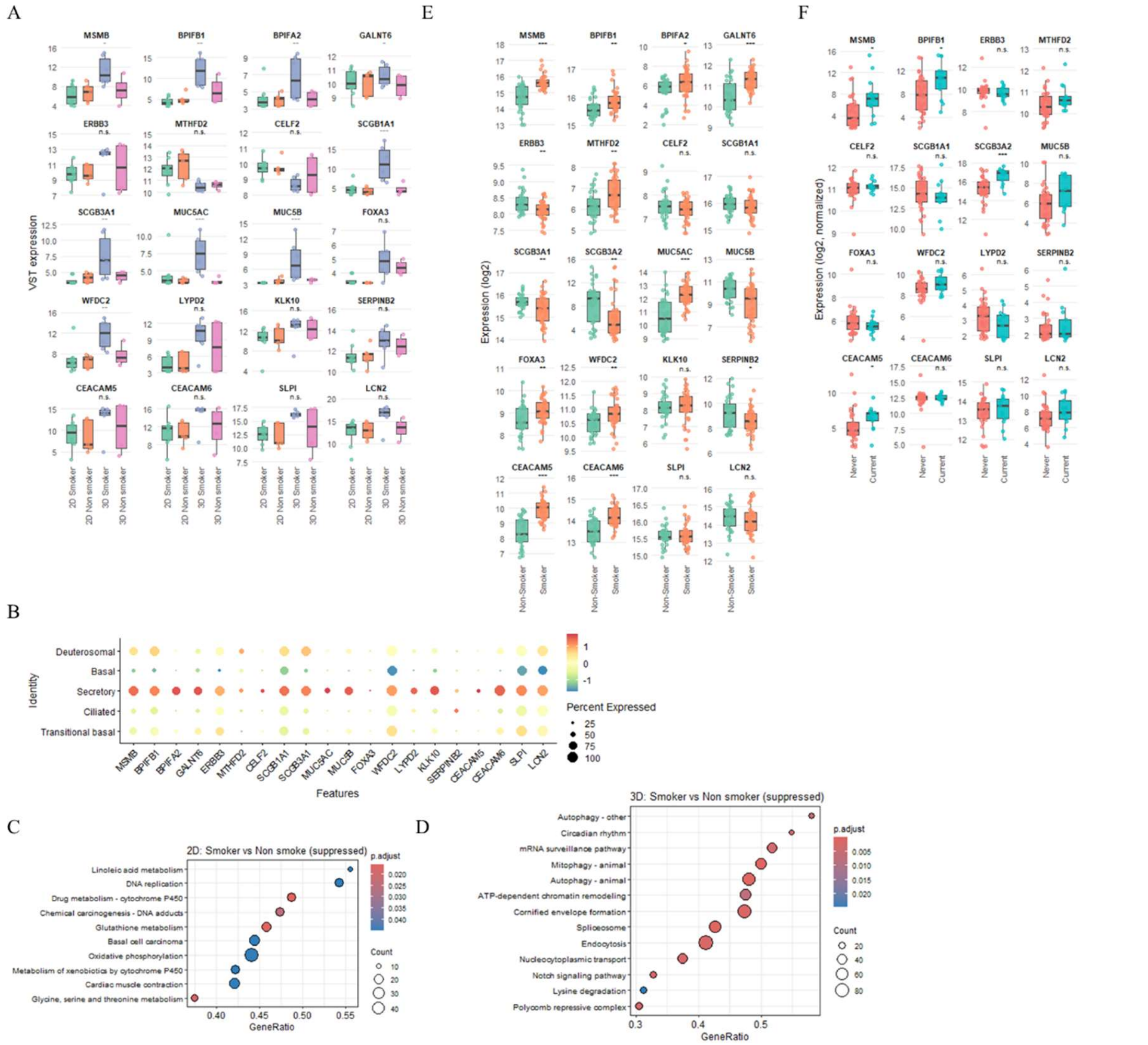

S6. Smoking imprints basal cells with a differentiation-dependent secretory program. (A) Expression of candidate secretory genes in basal cells and 3D organoids (DESeq2 Wald tests were used for pairwise contrast). (B) Dot plot of single-cell RNA-seq data from bronchial organoids showing expression of candidate genes across epithelial cell types. (C) KEGG over-representation analysis of genes downregulated in smoker versus non-smoker basal cells. (D) KEGG over-representation analysis of genes downregulated in smoker-derived versus non-smoker 3D organoids. (E) Validation of candidate genes in airway epithelial brush-biopsy dataset GSE34450. (F) Expression of candidate genes in whole-lung homogenates from the Lung Genomics Research Consortium dataset GSE47460. Asterisks indicate significance levels from Welch's t-test (* $p < 0.05$, ** $p < 0.01$, *** $p < 0.001$).

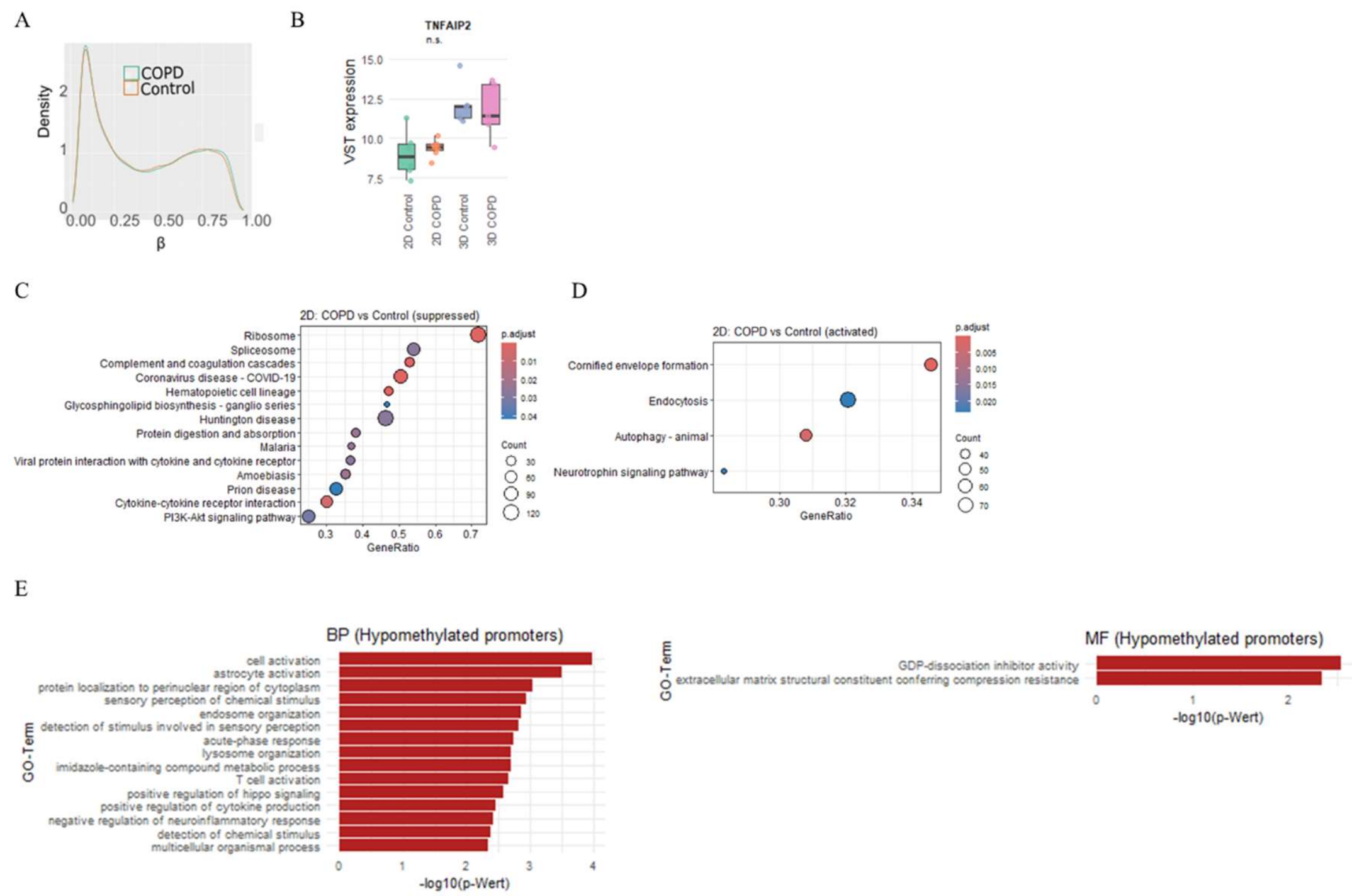

S7. Transcriptomic and promoter methylation changes in basal cells from COPD patients. (A) Density plot of promoter β-values in basal cells from control and COPD subjects. (B) Expression of *TNFAIP2* in basal cells and 3D organoids from control and COPD subjects. (C) KEGG over-representation analysis of genes downregulated in COPD. (D) KEGG over-representation analysis of genes upregulated in COPD. (E) GO for genes with hypermethylated and hypomethylated promoters.

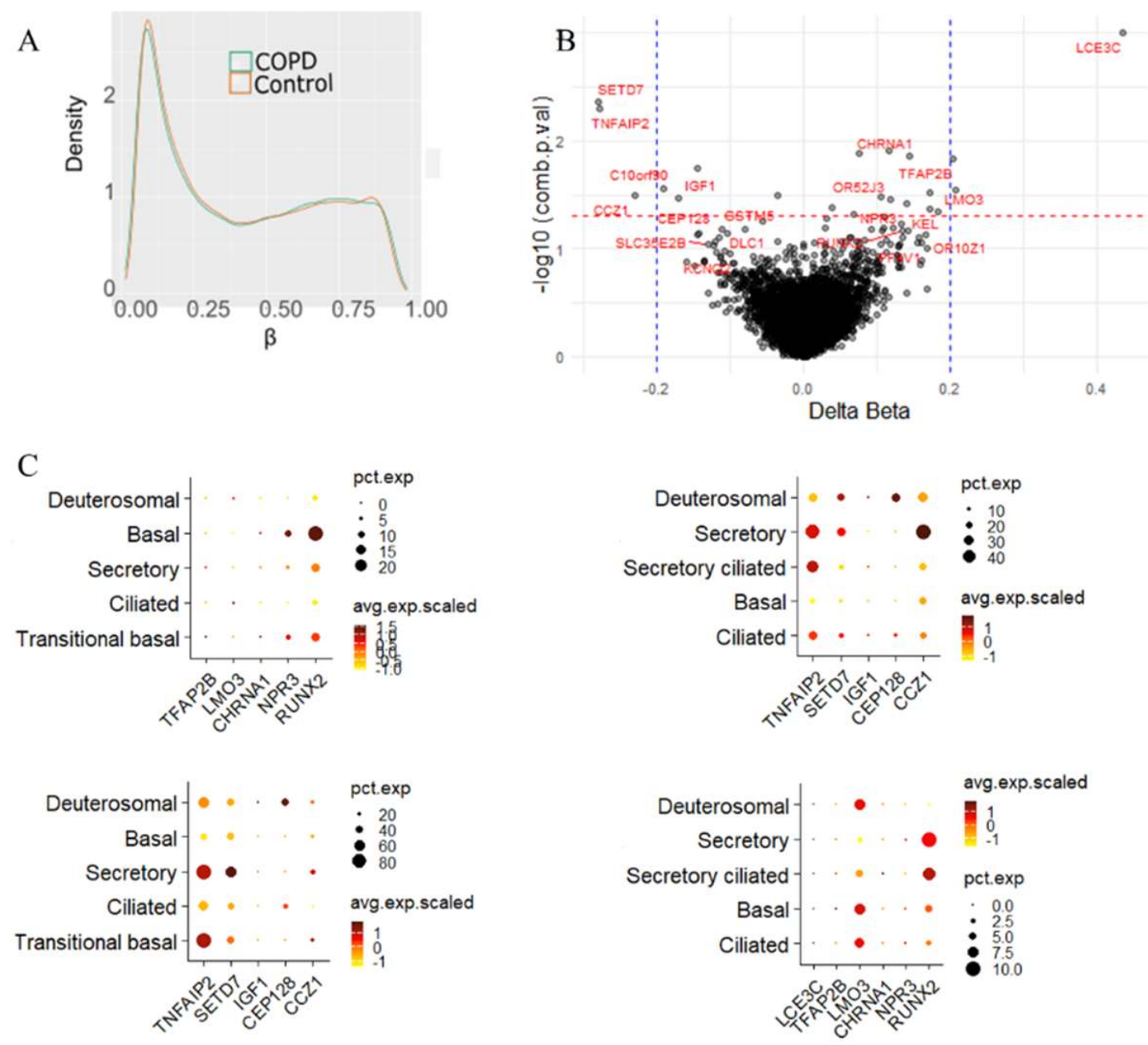

S8. Promoter methylation changes in COPD-derived organoids. (A) Density plot of promoter β-values in 3D bronchial organoids derived from control and COPD basal cells. (B) Volcano plot of promoter-associated methylation in COPD organoids highlighting hypomethylated and hypermethylated promoters. (C) Dot plots showing single-cell RNA-seq expression of candidate genes with altered promoter methylation across epithelial cell types in bronchial organoids and airway epithelium.
