# Supplementary material for: Current smoking and COPD are associated with differentiation-dependent secretory and inflammatory programs in airway basal cells
Source: Respir Res. 2026 Aug 29;27:343. doi: 10.1186/s12931-026-03847-4 (PMC13543530; doi:10.1186/s12931-026-03847-4)
Supplement: Supplementary file 2 [file 12931_2026_3847_MOESM2_ESM.pdf]

Current smoking and COPD are associated with differentiation-dependent secretory and inflammatory programs in airway basal cells

Felix Ritzmann, Michelle Brand, Gilles Gasparoni, Xuan Zhang, Frank Langer, Christian Herr, Yiwen Yao, Migdat Mustafi, Daniela Yildiz, Jörn Walter, Robert Bals, Christoph Beisswenger

ONLINE DATA SUPPLEMENT

Supplemental Methods:

Epigenetic Analysis

From each sample 700 ng of genomic DNA were bisulfite converted using the EZ DNA Methylation-Gold Kit (# D5006, Zymo research, USA) following the manufacturer's protocol, except the final elution volume was set to 10 µl. DNA methylation signatures were profiled using the Infinium MethylationEPIC V2.0 Kit (Catalog No. 20087708, Illumina, USA) according to the manufacturer's instructions and the arrays were scanned on an Illumina iScan platform. Raw data were processed using RnBeads (version 2.26.0). The data were mapped to the human reference genome hg38 (GRCh38), which provides up-to-date genomic coordinates for CpG sites, promoters, genes, and CpG islands. CpG sites and genomic regions, including genes and promoters, were considered, with methylation values aggregated as means across regions. Quality control included assessment of beta-value distributions, coverage histograms, boxplots, and SNP-based checks. Probes on sex chromosomes, low-coverage sites (<5×), CpGs in non-CpG contexts (CC, CAG, CAH, CTG, CTH, Other), and known SNP sites (minor allele frequency > 3 %) were excluded. Normalization was performed using the BMIQ method No background correction was applied (normalization.background.method = "none"). Quality control included removal of non-CpG contexts, SNP-affected CpG sites (SNP filter = 3), CpGs on sex chromosomes, and filtering of unreliable probes using the Greedycut algorithm with a detection P-value threshold of ≥ 0.05. In total, 59,081 probes and no samples were removed during quality control. Differential methylation analysis between groups was conducted using limma at the site level. Combined p-values (comb.p.val) were reported for promoters and genes. Statistical significance for promoter regions and genes was assessed using the combined p-value (comb.p.val), calculated across all CpG sites within each region.

Output tables (promoter, genes) were exported and processed in R for downstream statistical analysis. The promoter table were imported and sorted by the RnBeads comb.p.val statistic. For each promoter, mean methylation between two groups were extracted, and Δβ values (mean.mean.diff) were calculated. Gene-level annotation was added using org.Hs.eg.db (AnnotationDbi), and only entries annotated as *protein-coding* genes were retained for all analyses. To characterize the directionality of methylation changes, promoters were separated into hypomethylated ($\Delta\beta < 0$) and hypermethylated ($\Delta\beta > 0$) categories. Within each category, promoters were ranked by the combined rank, which reflects the aggregated RnBeads scoring metric. For visualization, a volcano plot was generated using ggplot2.

For KEGG enrichment [2, 3] the top 500 promoters of each category were selected. Gene symbols were mapped to Entrez Gene identifiers. KEGG over-representation analysis was performed separately for the hypomethylated and hypermethylated promoter sets using clusterProfiler::enrichKEGG (organism = "hsa", p-value cutoff = 0.05). Enrichment was tested using a hypergeometric model with the KEGG human background as reference. Pathways were ranked by nominal p-value, and results were visualized with the dotplot function. Gene Ontology (GO) enrichment analyses for differentially methylated promoter and gene regions were obtained from the corresponding RnBeads output.

To assess whether the top 100 genes of hyper- or hypomethylated promoters and genes identified (combined rank) from our epigenetic analysis were enriched in specific cellular clusters, we performed gene set enrichment analysis (GSEA) [4] using own single-cell RNA-seq data of organoids [5] and native proximal airway tissue (GSE168191/2) [1]. Differential expression analysis in secretory cells and gene-body-hypomethylated genes in ciliated cells were conducted using the FindMarkers function from the Seurat package [6], with thresholds of minimum 25% expression (min.pct = 0.25) and log fold change > 0.25 (logfc.threshold = 0.25). Genes were ranked according to their average log2 fold change in descending order to generate a ranked gene list. The top 100 hypo- or hypomethylated genes were used as a predefined gene set. GSEA was performed using the fgsea package with 10,000 permutations to assess the significance of gene set enrichment within the ranked list. Enrichment plots were generated to visualize the distribution of the hypomethylated genes across the ranked gene list, and leading-edge analysis identified genes contributing most to the enrichment signal. Gene expression signatures were

quantified using the AddModuleScore function implemented in Seurat [7, 8]. The resulting scores were projected onto the UMAP embedding using FeaturePlot to visualize expression patterns in transcriptomic space. Clusters annotated as secretory or ciliated cells were classified accordingly, whereas all remaining clusters were categorized as non-secretory or non-ciliated. Statistical differences in module scores between groups were assessed using the Wilcoxon rank-sum test.

Bisulfite-converted genomic DNA from basal cells was used to amplify promoter regions (~100 bp amplicons) with locus-specific primers. PCR products were subjected to targeted amplicon sequencing. Sequencing data were processed using a custom R script based on sangerseqR and Biostrings, in which reads were aligned to the corresponding reference amplicons and CpG methylation percentages were computed per site from the proportion of C (methylated) versus T (unmethylated) calls at each CpG position.

Single-cell RNA sequencing

Single-cell RNA-seq of airway organoids

For single-cell RNA sequencing (scRNA-seq), organoids from 10 wells derived from three donors were harvested by dissolving Matrigel in cold Cell Recovery Solution (Corning, #354253). Organoids were washed with cold PBS and dissociated into single cells using TrypLE Express (Gibco) with gentle resuspension for 15 min. After removal of TrypLE by washing in PBS, samples were multiplexed using the BD Human Single-Cell Multiplexing Kit (BD, #633781) according to the manufacturer's instructions. Cell number and viability were assessed using DRAQ7 (Thermo Fisher, #15106) and calcein (BD Biosciences, #564061). Single cells were captured on the BD Rhapsody platform, and cDNA and whole-transcriptome libraries were generated using the BD Rhapsody cDNA Kit (#633773) and WTA Amplification Kit (#633801) according to the manufacturer's protocol. Library concentration and quality were assessed using a Qubit Fluorometer (Qubit dsDNA HS Kit, #Q32851) and an Agilent 2100 Bioanalyzer (High Sensitivity DNA Kit, #5067-4626). Libraries were sequenced on an Illumina NovaSeq 6000 platform at a depth of approximately 50,000 reads per cell.

Raw FASTQ files were processed using the BD Rhapsody WTA Analysis Pipeline on the Seven Bridges platform and aligned to the human reference genome GRCh38. Downstream analysis was performed in R using Seurat v4.0. Cells classified as multiplets or undetermined by the BD pipeline were excluded. Additional quality filtering removed cells with fewer than 1,000 detected genes or more than 50% mitochondrial transcripts. The filtered dataset was normalized using Seurat's NormalizeData function (scale factor 10,000) and scaled using ScaleData. Datasets from two cohorts were integrated using the Seurat integration workflow. Cell clustering was performed using a shared nearest neighbor (SNN) graph-based approach with a clustering resolution of 0.3, and UMAP was used for nonlinear dimensionality reduction and visualization. To assess sample representation across the integrated dataset, UMAPs were inspected by sample identity prior to annotation. Cluster identities were assigned based on the expression of canonical airway epithelial marker genes visualized by DotPlot and UMAP inspection. *KRT5* and TP63 were used to identify basal and transitional basal populations; *SCGB1A1*, *MUC5B*, and *MUC5AC* to identify secretory populations; and *CDC20B*, *E2F7*, and *FOXJ1* to identify deuterosomal and ciliated populations. Based on these marker profiles, clusters were annotated as basal, transitional basal, ciliated, secretory, and deuterosomal.

Reanalysis of public proximal airway scRNA-seq data

Publicly available scRNA-seq data from native human proximal airway tissue (GSE168191/2) were reanalyzed using Seurat. Datasets from donor 3 proximal airway tissue were imported from .h5 files and converted into Seurat objects. Cells were filtered to retain those with more than 200 and fewer than 8,000 detected genes and less than 15% mitochondrial transcripts. After normalization (NormalizeData, scale factor 10,000), identification of 2,000 variable genes (FindVariableFeatures, method = vst), and scaling (ScaleData), principal component analysis (PCA) was performed. Cells were clustered using a shared nearest neighbor graph (FindNeighbors (dimensions 1–10), FindClusters (resolution 0.3)) and visualized by UMAP. Clusters were annotated based on canonical marker gene expression using DotPlot. Based on the expression of epithelial and lineage markers, epithelial populations were distinguished from immune, stromal, and endothelial cells, and the epithelial subset was extracted for further analysis. The epithelial subset was then reclustered after repeat scaling and PCA using FindNeighbors (dimensions 1–10), FindClusters (resolution 0.4), and RunUMAP. Final annotation identified basal, secretory, secretory-ciliated, ciliated, and deuterosomal epithelial states.

Bulk

Total RNA concentration and purity were assessed using a Nanodrop 8000 spectrophotometer (Thermo Fisher Scientific, USA). Libraries were prepared for bulk mRNA sequencing and run on a DNBSeq PE150 platform (20 million paired-end reads per sample; BGI Tech Solutions). Sequencing reads were aligned to the human GRCh38 reference genome using STAR (version 2.7.3a) with default parameters, and gene-level count tables were generated with featureCounts (version 2.0.1) using default settings and gene IDs as annotation keys.

Bulk RNA-seq count matrices from two sequencing runs were processed in R using DESeq2 [9, 10]. Ensembl gene identifiers were cleaned and mapped to HGNC symbols using biomaRt, and genes without unique mappings were removed. Both sequencing runs were harmonized by intersecting shared genes and merged column-wise. Metadata tables were merged accordingly, and the experimental variables Smoke or COPD were encoded as a four-level factor (Yes_2D, No_2D, Yes_3D, No_3D). DESeq2 datasets were generated and lowly expressed genes were filtered. Differential expression was tested using DESeq2 (negative binomial GLM) with Wald tests. Log2 fold changes were shrunken using apeglm. P values were adjusted for multiple testing using the Benjamini–Hochberg procedure across genes, and significance was indicated on boxplots of VST-transformed expression. KEGG over-representation analysis for the contrasts was performed using cluster profiler [2]. Differentially expressed genes were mapped from HGNC symbols to Entrez Gene identifiers, and the analysis background was defined as all genes with valid Entrez IDs in the DESeq2 results. The test set consisted of significantly regulated genes ($padj \leq 0.05$) with an absolute shrunken $\log_2$ fold change ≥ 0.58, corresponding to approximately 1.5-fold regulation. Enrichment was computed using a hypergeometric test with Benjamini–Hochberg correction (p-value cutoff = 0.05, q-value cutoff = 0.2). Resulting KEGG pathways were ranked by adjusted p-value and visualized as dot plots using standard cluster profiler functions.

Published microarray data from GSE34450 [11] and GSE47460 (Lung Genomics Research Consortium, platform GPL14550) were obtained from GEO and processed uniformly. For GSE34450, expression matrices were extracted from the Series Matrix file and $\log_2$-transformed when raw intensities indicated non-log scaling. Probe annotations were retrieved from the hgu133plus2.db package, and Affymetrix probe IDs were mapped to HGNC gene symbols using AnnotationDbi. Smoking status was extracted from sample metadata, harmonized into two groups (Smoker and Non-Smoker), and samples without a clearly defined status were excluded. For GSE47460, only samples profiled on GPL14550 were included, and the provided $\log_2$-normalized expression values and phenoData disease state and smoker? were used to derive disease labels and smoking categories; samples annotated as Interstitial lung disease were excluded, and only subjects with “1-Current” or “3-Never” were retained and recoded as Current

and Never smokers, respectively. Probes lacking an assigned gene symbol were removed, and for genes represented by multiple probes, expression values were aggregated by the mean across probes. For a predefined panel of candidate genes, expression differences between smoking groups were assessed using two-sided Welch's t-tests and visualized by boxplots.

1. Basil MC, Cardenas-Diaz FL, Kathiriya JJ, Morley MP, Carl J, Brumwell AN, Katzen J, Slovik KJ, Babu A, Zhou S, et al: **Human distal airways contain a multipotent secretory cell that can regenerate alveoli.** *Nature* 2022, **604:**120-126.
2. Yu G, Wang LG, Han Y, He QY: **clusterProfiler: an R package for comparing biological themes among gene clusters.** *OMICS* 2012, **16:**284-287.
3. Kanehisa M, Goto S: **KEGG: kyoto encyclopedia of genes and genomes.** *Nucleic Acids Res* 2000, **28:**27-30.
4. Korotkevich G, Sukhov V, Budin N, Shpak B, Artyomov MN, Sergushichev A: 2021.
5. Brand M, Kempen P, Risch T, Guedes M, Bals R, Ritzmann F, Yildiz D, Beisswenger C: **IL-13 drives a mucin phenotype: insights from a human apical out airway organoid model.** *European Respiratory Journal*, **64:**PA2670.
6. Hao Y, Hao S, Andersen-Nissen E, Mauck WM, 3rd, Zheng S, Butler A, Lee MJ, Wilk AJ, Darby C, Zager M, et al: **Integrated analysis of multimodal single-cell data.** *Cell* 2021, **184:**3573-3587 e3529.
7. Butler A, Hoffman P, Smibert P, Papalexi E, Satija R: **Integrating single-cell transcriptomic data across different conditions, technologies, and species.** *Nat Biotechnol* 2018, **36:**411-420.
8. Stuart T, Butler A, Hoffman P, Hafemeister C, Papalexi E, Mauck WM, 3rd, Hao Y, Stoeckius M, Smibert P, Satija R: **Comprehensive Integration of Single-Cell Data.** *Cell* 2019, **177:**1888-1902 e1821.
9. Love MI, Huber W, Anders S: **Moderated estimation of fold change and dispersion for RNA-seq data with DESeq2.** *Genome Biol* 2014, **15:**550.
10. Cunningham F, Allen JE, Allen J, Alvarez-Jarreta J, Amode MR, Armean IM, Austine-Orimoloye O, Azov AG, Barnes I, Bennett R, et al: **Ensembl 2022.** *Nucleic Acids Res* 2022, **50:**D988-D995.
11. Wang G, Xu Z, Wang R, Al-Hijji M, Salit J, Strulovici-Barel Y, Tilley AE, Mezey JG, Crystal RG: **Genes associated with MUC5AC expression in small airway epithelium of human smokers and non-smokers.** *BMC Med Genomics* 2012, **5:**21.
